# Complex Distribution Phenomena and Plastic Binding of Test Chemicals in Cell Culture Experiments: Exemplification by Tebufenpyrad

**DOI:** 10.3390/ijms27125547

**Published:** 2026-06-19

**Authors:** Mahshid Alimohammadi, Hiba Khalidi, Elias Zgheib, Anna-Katharina Holzer, Naja Bürgers, Céline Brochot, Patrik Lundquist, Viktoria Magel, Baiba Gukalova, Edgars Liepinsh, Marcel Leist

**Affiliations:** 1 In Vitro Toxicology and Biomedicine, Department Inaugurated by the Doerenkamp-Zbinden foundation, University of Konstanz, 78457 Konstanz, Germany; mahshid.alimohammadi@uni-konstanz.de (M.A.); anna-katharina.holzer@uni-konstanz.de (A.-K.H.);; 2 Certara Predictive Technologies, Level 2-Acero, 1 Concourse Way, Sheffield S1 2BJ, UK; hiba.khalidi@certara.com (H.K.); elias.zgheib@certara.com (E.Z.); celine.brochot@certara.com (C.B.); 3 Department of Pharmacy, Uppsala University, SE-751 23 Uppsala, Sweden; patrik.lundquist@uu.se; 4 Latvian Institute of Organic Synthesis, LV-1006 Riga, Latvia; baiba.gukalova@osi.lv (B.G.); ledgars@farm.osi.lv (E.L.); 5 Center for Alternatives to Animal Testing in Europe (CAAT-Europe), University of Konstanz, 78457 Konstanz, Germany

**Keywords:** biokinetics, plastic binding, NAM, in vitro testing, neurotoxicity, mitochondria

## Abstract

Biokinetic complexities (plastic sorption, protein binding, and cellular accumulation) may cause large discrepancies between nominal and biologically effective concentrations of test compounds assessed by new approach methods (NAMs). This case study was performed to explore a generally applicable workflow that addresses biokinetic complexities in the context of NAM-based hazard testing for next-generation risk assessment (NGRA). The pesticide tebufenpyrad (TEBU) is a challenging test compound, as it (i) is hydrophobic, (ii) has an intracellular target (mitochondrial respiration), and (iii) is acting at low concentrations (susceptible to biokinetic complexities). In the newly established NeuriTox-M neurotoxicity assay, based on human dopaminergic (LUHMES) neuron cultures, TEBU showed toxic effects at 20 nM. Mass spectrometric analyses of various experimental setups showed that a large fraction (75% to >90%) of TEBU was adsorbed to plastic. This effect was strongly attenuated by albumin in the medium. Cells, cultured on plastic, were considered unsuitable to assess cellular uptake. Therefore, alternatives were explored: when cells were used as suspension cultures (3% *v*/*v*) in albumin-containing medium, analysis worked best. Under such conditions, the concentration ratio (cells/medium) of TEBU was around 10. Data from an in vitro distribution (VIVD) model were in good agreement with the measurements. VIVD predicted the unbound medium TEBU concentration (C_u_) to be 2–3 orders of magnitude below the nominal concentration and the total cellular concentration to be 10–100-fold above. Standard cell culture assays showed that the medium albumin content indeed altered the TEBU toxicity threshold. More such studies are needed to embed biokinetics information into NGRA.

## 1. Introduction

The actual cellular concentration of a test chemical may differ from the nominal concentration in many cell culture setups used for toxicological tests [1,2]. The nominal concentration is a theoretical value, under the assumption that the test chemical distributes equally in all cell culture compartments and is not lost. The actual cellular concentration can either be determined by measurements or by complex prediction models [3,4,5]. These need to account for compound losses due to plastic binding, evaporation, degradation, and cellular metabolism. In addition, they need to consider protein binding, lipid binding, and distribution phenomena, in addition to cell volume and cell composition [1,2,6].

To use data from cell culture models for toxicological predictions, it is important to obtain good estimates (by modeling or measurement) of test compound concentrations in the relevant culture compartments. The two major reasons are that (i) this knowledge is required to compare data between two different test methods, also termed new approach methodologies (NAMs) [7], and that (ii) this information is also required to extrapolate NAM data to relevant in vivo doses by in vitro-to-in vivo extrapolations (IVIVEs) [8,9,10]. More precisely, not just the real total concentrations should be known, but also the free fraction (f_u_), i.e., the biologically available fraction of a chemical [11,12].

IVIVE usually starts with a NAM-based point-of-departure (PoD), i.e., a test compound threshold concentration determined by NAM testing. This PoD should not only be a theoretical value, but it is ideally reflecting the real free concentration at the target site of the toxicant. In many cases, this requires knowledge of intracellular concentrations [6,13,14]. Differences in the expression of transport proteins between cell models and in vivo tissues can also result in unrepresentative intracellular concentrations, even when external free concentrations appear equivalent [2]. These mechanisms may lead to bioavailable concentrations that differ strongly from nominal levels, leading to misleading assumptions about potency [1,12].

IVIVE connects in vitro toxicity data to realistic human exposure scenarios [15,16] by translating in vitro concentration–effect relationships into realistic in vivo exposure scenarios [12,17]. To ensure that this link is reliable, free concentrations in in vitro systems should be considered. A common simplification in IVIVE is to estimate f_u_ using lipophilicity data (e.g., logP). This approach fails to capture critical structure-specific factors, such as charge distribution, flexibility, or steric parameters.

One option, to reduce uncertainty and to improve the predictive value of in vitro assays, is to experimentally measure total and free concentrations, or to improve models by accounting for protein binding, plastic sorption, and cellular uptake [2,17]. This provides a more robust foundation for interpreting in vitro data and enhances the accuracy of toxicological risk assessments [18,19]. Studies have shown that effect concentrations based on measured free concentrations correlate more closely with in vivo toxicity data than those based on nominal concentrations [20].

Some test compounds may bind to protein in cell culture media to such an extent that toxic effects are reduced [21]. Albumin, the most abundant protein in blood plasma and a supplement in culture media, exhibits a high affinity for hydrophobic molecules, thereby significantly limiting their availability for cellular uptake and toxic action. Increased bovine serum albumin (BSA) concentrations in culture media may elevate EC_50_ values, even for chemicals with relatively low BSA affinity [2,22].

The importance of protein binding has been shown for the drug chlorpromazine: In the presence of 600 µM BSA (physiological concentrations), the plastic binding of the drug was reduced, and 94% was bound to albumin, leaving only 6% as free, bioavailable test compound. Such strong protein-binding behavior must be carefully considered when extrapolating bioactivity data for lipophilic drugs [23] measured in NAM with low protein content to the human situation, where albumin concentrations are high.

A similar principle applies to lipid and plastic binding. Lipophilic chemicals tend to have inhomogeneous distributions in cell cultures as they bind to lipids and plastics in addition to proteins. When a big fraction of the compound attaches to the plastic, its available free concentration is reduced. Moreover, compounds with a high lipophilicity (large logP) tend to accumulate in the cells because of the high affinity to lipids. For example, over 50% of cypermethrin (logP ~6.6) binds to plastic, and about 15% is absorbed by cells in a rainbow trout gill cell line [24]. Similarly, polychlorinated biphenyls (PCBs) exhibit a strong affinity for plastics. In a study on neural crest cells, about 75% of PCBs were bound to the cell culture dish plastic after 24 h [25]. Such effects should be considered for the exposure and risk assessment of these compounds. Also, for amiodarone (AMI) (logP ~7.57) a 40% loss by plastic binding has been observed [26]. Such binding behaviors must be considered when interpreting in vitro neurotoxicity results.

In another study, Palmgrén et al. [27] demonstrated that basic, lipophilic drugs such as propranolol and midazolam adsorb significantly to polystyrene surfaces under in vitro conditions, with only 32% and 24% of the initial concentrations remaining in aqueous solution, respectively. Drug retention in cells was also notable, with 44% of propranolol and 24% of midazolam accumulating in the cells. Interestingly, the study found that higher drug concentrations resulted in proportionally lower plastic adsorption, suggesting that polystyrene surfaces have a limited number of binding sites [27]. In contrast, compared to highly lipophilic compounds, hydrophilic and moderately lipophilic substances, including ibuprofen, demonstrate less binding to plastic surfaces and proteins [28].

The growing reliance on in vitro models in pharmacology and toxicology [29] has revealed key challenges in quantifying chemical concentrations [1]. Biokinetic considerations are particularly important at low nominal concentrations, i.e., for potent toxicants, such as tebufenpyrad (TEBU).

LUHMES (Lund human mesencephalic) cells are a robust in vitro model for studying toxicity to dopaminergic neurons. They have been derived from human fetal mesencephalic tissue and can be driven to differentiate into fully post-mitotic neurons [30,31]. They grow long neurites (often exceeding 500 µm) and they express functional voltage-gated ion channels as well as several dopaminergic markers, including tyrosine hydroxylase and the dopamine transporter. These cells are highly sensitive to neurotoxicants such as 1-methyl-4-pyridinium (MPP^+^), a mitochondrial complex I inhibitor linked to Parkinson’s disease [30]. They can be switched to a predominantly mitochondrial energy metabolism in media containing a low glucose concentration [32].

TEBU is a plant protection product (insecticide of the methyl pyrazol class). Its inhibition of mitochondria [33] has raised concerns for human safety, because compounds with a similar target (complex I of the mitochondrial respiratory chain) trigger the AOP: 3 [34] adverse outcome pathway [35] that leads to the degeneration of dopaminergic neurons, and to parkinsonian motor deficits.

The support of TEBU risk assessment by data from cultured human dopamine neurons requires knowledge on TEBU’s intracellular concentration. Use of a PoD based on nominal concentrations is likely to differ from a ‘real’ PoD, based on the actual cellular concentration. This may lead to erroneous estimates of the pesticide’s potential risks. As TEBU is a highly lipophilic pesticide (experimental logP: ~4.93) [36], experimental studies on cellular concentrations are warranted.

As currently few studies describe the assessment of intracellular concentrations of neurotoxicants, more work is required on sampling strategies to provide guidance on data interpretation. We used TEBU as an exemplary compound to measure test chemical distribution in LUHMES neuronal cultures. We aimed to explore how bioactivity data from NAM may be combined with measurements of the test compound distribution to provide a realistic PoD for risk assessment. Moreover, we compared measured data with predictions from the Virtual In Vitro Distribution (VIVD) [4] model to explore whether the higher granularity of model predictions (e.g., on cell compartments or on total vs. unbound medium concentrations) may expand the information provided by analytical methods. By using TEBU as a challenging test compound (high potency and lipophilicity), we explored potential shortcomings and improvement strategies for biokinetics data production. We also focus on the particular challenges of neurotoxicity: In commonly used two-dimensional neural cultures, the cellular volume is very small compared to the volume of cell culture medium. With potent toxicants, this results in very little compound within cells to be analyzed. With highly potent compounds this also means that losses to plastic may affect available concentrations to a large degree. Our study demonstrates how this issue may be overcome by the use of cell suspensions, and it highlights various problems of calibration and compound handling to pave the way towards a broader use of biokinetics studies in next-generation risk assessment (NGRA).

## 2. Results

### 2.1. Selective and Potent Neurotoxicity of TEBU

**Figure 1:**
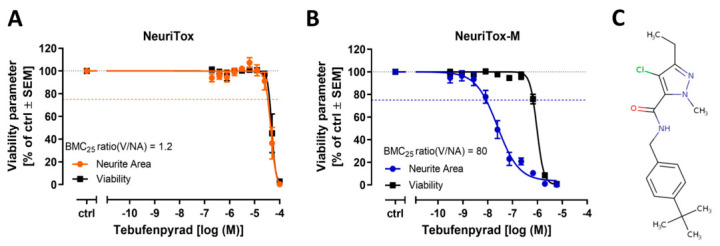
The effects of TEBU on the neurite area and cell viability of developing dopaminergic neurons. LUHMES cells were exposed to TEBU from d2 to d3 of differentiation for 24 h. Two assay conditions were used. In the NeuriTox test (**A**), standard medium was used. In the NeuriTox-M assay (**B**), glucose in the medium was substituted with galactose. Following treatment, the cells were stained with calcein-AM plus Hoechst H-33342. The neurite area (NA) and cell viability (V) were quantified using an automated image analysis algorithm. They were normalized to the DMSO (0.1%) vehicle control (ctrl). Data represents means ± SEM from three independent experiments. Benchmark concentrations for 25% effect (BMC_25,_ second dash line on the Y axis) were calculated for each parameter, and BMC_25_ ratios (V/NA) for neurite effects vs. viability are given. Additional details for inter-operator reproducibility are provided in Figure S1. (**C**) Tebufenpyrad’s chemical structure is shown [36].

Tebufenpyrad, although being a specific inhibitor of the mitochondrial respiratory chain complex I (cI) [37], did not trigger specific neurotoxicity in LUHMES cells [33]. This contrasts with findings on many similarly acting compounds, such as MPP^+^, rotenone or deguelin [33,38,39]. It was suggested earlier that specific effects of cI inhibitors may be observed in LUHMES neurons by running the NeuriTox assay in medium, in which glucose is replaced by galactose [32,40]. Under such conditions, neurons become more dependent on their mitochondrial activity (M) [41]; therefore, we coined the term NeuriTox-M for this new assay variant [42,43]. Here, we compared the readouts for TEBU between the NeuriTox and the NeuriTox-M assay (see Figure 1 and Figure S1): TEBU showed a low potency (BMC_25_ ≈ 40 µM) in the NeuriTox assay, and it triggered cell death without any specific effects on neurites. This may appear astonishing, given that TEBU blocks mitochondrial respiration in the 30–50 nM range [37,44]. The explanation is that LUHMES have a very powerful glycolytic machinery [45], and they are able to fully maintain ATP supply upon stress, as long as glucose supply is sufficient [32,33]. When examined in the NeuriTox-M assay, TEBU actually showed a much higher potency. The BMC_25_ for neurite effects was 20 nM (>1000-fold lower than under NeuriTox conditions).

Moreover, selective toxicity was observed, as neurite damage occurred at concentrations approximately 80× lower than those leading to cytotoxicity. These data are well in line with earlier findings on complex I inhibitors [32,41]. To further demonstrate the robustness of this effect, additional studies were included. Similar data were consistently obtained over several years and by three additional operators, corroborating the reliability of the NeuriTox-M assay (Figure S1).

The data from the NeuriTox-M assay suggest that TEBU has a much higher neurotoxic hazard potency and damage selectivity than assumed earlier. Such findings provide the background rationale for further studies on the compound, as this environmental toxicant may pose a risk for human health. The prediction of human neurotoxicity from cell-based studies is a particular challenge for hydrophobic compounds (high logP), such as TEBU. It has been recognized that a major difficulty in the evaluation of such compounds is a correct prediction of the fraction that reaches the brain. Moreover, it is often unclear whether potential accumulation in cells has to be factored in [46,47]. The following study is a first attempt (scoping study) on how this may be addressed, and which problems are encountered during such studies.

### 2.2. Preparation of Working Stocks to Assess Plastic Binding and Cell Uptake

This study aimed not only at understanding the distribution behavior of TEBU in cell cultures, but it also explored methods to approach this and potential pitfalls of common laboratory techniques. Therefore, a set of pilot experiments were performed to establish analytical methods and to explore suitable test conditions. Based on experience from these tests, it became clear that plastic binding required attention, not only for cell experiments, but also already during the preparation of stock solutions. Therefore, a very careful procedure was used to prepare TEBU working stocks in the medium. Moreover, separate stocks were prepared with and without the addition of albumin (1% BSA) to assess the influence of protein on the behavior of TEBU in cell culture experiments (Figure S2).

We also took care to produce reference gold standards to quantify all other samples, including the stock solutions. We assumed that plastic binding and other losses would not occur for the handling of TEBU in pure DMSO. To avoid any additional plastic contact, the gold standard calibration solutions (CS1, CS2) were prepared directly within the sample block (Figure S3).

**Figure 2:**
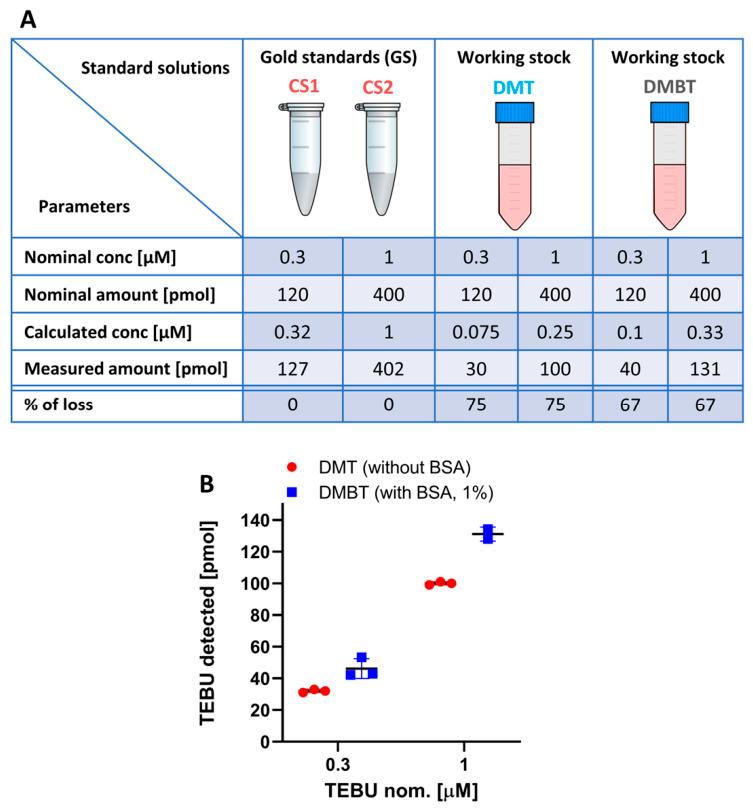
The recovery of TEBU from ‘working stocks’. Working stocks (DMT and DMBT) were prepared by diluting the 1 mM TEBU master stock in DM or DMB to a final concentration of 1.33 µM. These solutions were made in 50 mL polypropylene conical centrifuge tubes (see Figure S2). To obtain 200 µL solutions with nominal concentrations of 0.3 µM and 1 µM, working stocks were diluted with either DM or DMB within the sample block. To calibrate the final data, gold standard samples (CS1 and CS2) were prepared by directly diluting calibration solutions (CS 10 µM or 3.3 µM TEBU in DMSO) inside the sample block with differentiation medium (DM) (see Figure S3). TEBU concentrations of CS1 and CS2 were quantified by LC-MS, based on the peak area ratio to the internal standard (diclofenac). For comparison across samples, the detected amounts of TEBU in gold standards were normalized to an equivalent of 400 µL total volume, reflecting the initial volume used in other test conditions. (**A**) These values were used for constructing calibration curves and for calculating TEBU concentrations and recovery across all experimental conditions. (**B**) Working stocks were measured (repeated experiments) and quantified, using gold standard calibration curves. Data are displayed as the actual amount measured. Data in (**A**) allowed a comparison with the nominal (expected) amount. Note that a substantial loss was observed in the working stock samples prepared in 50 mL tubes (despite precoating), with only 25% recovery in DMT. There was an improved recovery of 33% in DMBT which shows the effect of BSA supplementation of working stocks. The percentage of loss is given in (**A**) relative to nominal concentrations.

After the construction of a calibration curve (based on peak areas of CS1 and CS2 and their known concentration), the actual amount of TEBU in the working stocks was determined. These measurements showed that the relative quantification worked well: solutions of 0.3 µM and 1 µM showed the correct proportion of detection signals. It also became clear that more TEBU was recovered from BSA-containing medium (DMBT) compared to the medium without BSA (DMT) (Figure 2).

A quantitative approach, relating the recovered amount of TEBU to the nominal concentration, showed that the recovery in DMT was 25% (corresponding to a loss of 75%). The recovery in DMBT was 33%, corresponding to a loss of 67% (Figure 2). The losses were highly consistent across all experiments, and the most likely explanation is the binding of TEBU to the plastic of the working stock vessels [47,48].

Even in the light of the literature on plastic adsorption of some compounds [27], the losses appeared very high. To provide additional evidence, we therefore conducted an entirely different study with other operators, analytics, and experimental design. The final goal was again to estimate potential losses due to plastic binding: Stock solutions of TEBU and of the related compound, tolfenpyrad, were prepared in 50 mL tubes as described before. The medium was either protein-free or contained 0.5% human serum albumin (HSA). The recovery in the protein-containing medium was complete (100%, within the normal experimental variation). In serum-free medium >90% of the compounds were lost (Figure S4). These experiments confirm the key finding of strong losses in medium with no (or low) protein. The medium with HSA reduced compound losses under these experimental conditions. Another set of experiments was also performed in a slightly altered setting (1.5 mL microfuge tubes instead of 50 mL tubes). Also here, virtually all compound was depleted (most likely by plastic binding), while HSA prevented such losses [49]. This series of experiments thus confirmed the extreme tendency of TEBU (or related compounds) to stick to plastic, and it showed that protein in the medium attenuates (or abolishes) such losses (Figure S4) [50,51].

### 2.3. TEBU Distribution in Adherent Cell Cultures

**Figure 3:**
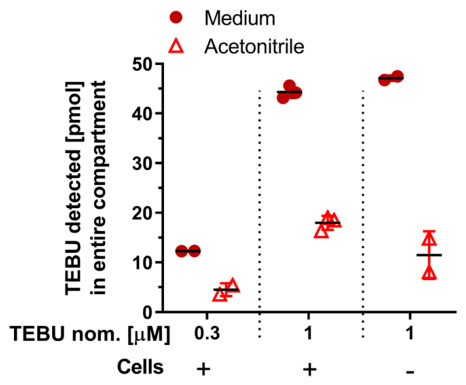
The recovery of TEBU from cell culture compartments in the standard LUHMES cell culture system. LUHMES cells (d2) were cultured at a density of 220,000 cells/cm^2^ (altogether 400,000 cells per well, with a total cell volume of 0.56 µL) in 24-well polystyrene flat base cell culture plates. After 24 h, the medium was replaced with 400 µL DMT (nominal concentrations were as indicated under the *x*-axis). After an incubation time of 30 min, the medium was collected and transferred into the sample block. Then 200 µL acetonitrile was added to the cell layer. The plate was shaken for 30 min to lyse cells and to extract cell-associated and plastic-bound TEBU, and then an aliquot of this solution was transferred to the sample block. The same procedure was done in parallel for wells without cells (−). The detected amount of the TEBU, indicated on the *y*-axis, corresponds to the initial total sample volume for each compartment. Experiments were performed 2–3 times and the horizontal black bars show the averages ± the range. A recovery overview is given in Figure S5.

After the initial experiments, we explored the distribution behavior of TEBU in standard cell cultures (adherent LUHMES neurons). Standard medium (DMT), as used for toxicity testing in NeuriTox assay, was used. After exposure of the cultures to TEBU (0.3 and 1.0 µM, nominal), its actual amount was determined in the supernatant medium. The fraction bound to plastic was analyzed after the removal of all medium and rinsing of cells and plastic with acetonitrile. About 75% of the recovered TEBU was found in the medium, and 25% were in the acetonitrile fraction [52]. Both fractions increased proportionally with the amount of TEBU added, and the data were highly reproducible across experiments (Figure 3). Thus, plastic binding was proportional to the amount added. The total mass balance was 60% (Figure S5a,b), and was judged satisfactory for the objective of the experiment; losses may have occurred during the preparation and pipetting steps required to prepare.

When the same experiment was performed without cells (with 1.0 µM TEBU, nominal), similar amounts were recovered in medium and rinse fractions (no significant difference between conditions with and without cells; Figure 3 and Figure S5c). We concluded from these data that measurements of TEBU within cells (using the difference in the ‘bound fraction’ with and without cells) would be unreliable under these conditions. The requirement of resources (cells, time, and analytical effort) provided to achieve clear results with this approach was considered too high. A key problem, considered hard to overcome, is that cells (0.56 µL volume) contributed only 0.14% of the total experimental volume (with 400 µL medium). On the positive side, one may additionally conclude that experiments aimed at identifying some basic chemical behavior (such as plastic binding) may be performed in a simplified setup without cells.

### 2.4. Setup of Assay Conditions for Suspension Cultures

**Figure 4:**
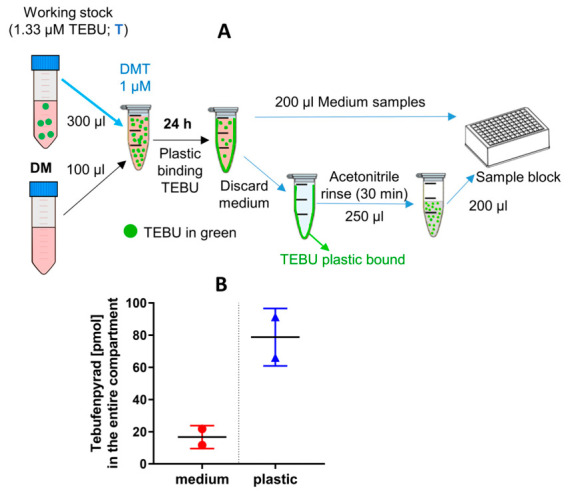
Sample preparation to assess TEBU binding to plastic vials. (**A**) A schematic of the experimental procedure used to assess TEBU adsorption to the plastic surface. To prepare the samples, 300 µL of DMT, containing a nominal concentration of 1.3 µM TEBU (the actual amount was 100 pmol according to Figure 2), was diluted in 1.5 mL microfuge tubes by adding 100 µL of DM. The final concentration was nominally 1 µM. After 24 h incubation at room temperature, the medium was collected, and the tubes were rinsed with 250 µL acetonitrile for 30 min to extract plastic-bound TEBU. (**B**) TEBU amounts in medium and in the rinse (plastic-bound fraction) were quantified. Data are expressed in picomoles (pmol) per fraction. The detected amount of TEBU, indicated on the *y*-axis, corresponds to the total volume at the start of the experiment (400 µL). The amount of TEBU actually present in the experiment was 100 pmol (Figure 2). Based on this, the mass balance in the experiment (plastic + medium) was close to 100%. Additional data for the confirmation of extensive compound losses by storage in plastic vessels is provided in Figure S4.

As an alternative to measurements in adherent cultures, we considered the use of suspension cultures (Figure S6). This approach offers two advantages: (i) the relative cell volume (cells vs. medium) can be increased under such conditions; and (ii) plastic binding can be analytically separated from cell uptake [53,54]. In a pilot experiment, we tested a potential setup, without cells. Medium containing TEBU was added to 1.5 mL Eppendorf microfuge tubes (1.0 µM TEBU, nominal), left for 24 h, and then the amounts of test compound in the medium versus ‘bound to plastic’ were assessed (Figure 4A).

The total recovery was 100%, with three quarters of TEBU bound to plastic and one quarter remaining in the medium (Figure 4B). Note that the tube’s plastic properties (polypropylene) differ from those of 24-well plates (polystyrene). Although plastic binding was high, we considered the remaining amount sufficient for all uptake experiments. The high amount of plastic binding was considered acceptable, as it was in a different fraction than in the medium (or in the cells that may be suspended therein).

**Figure 5:**
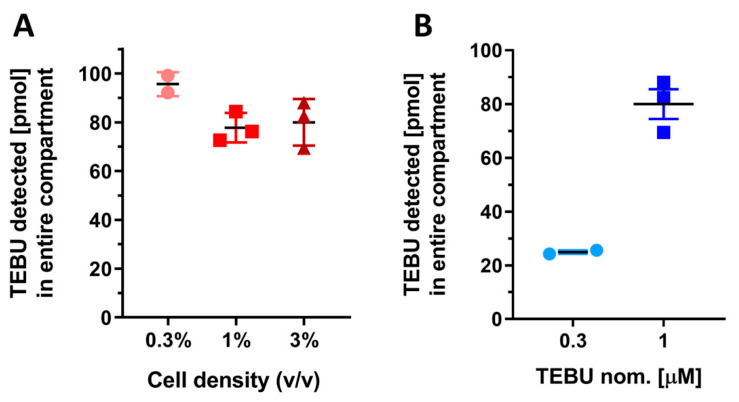
The quantification of TEBU in LUHMES cell suspensions of varying cell densities. LUHMES cell suspensions (in 400 µL DMBT) of 0.3%, 1%, and 3% *v*/*v* were generated in 1.5 mL microfuge tubes in DMBT. After 30 min of incubation, 200 µL of the cell suspension (containing both medium and cells) was collected and transferred to a sample block for analysis. Note that the incubation time is based on extensive pilot studies that showed that equilibrium distribution is reached within <5 min. An exposure time of 30 min was chosen for practical reasons of sample preparation. (**A**) Amount of TEBU detected. (**B**) Cell suspensions (3% *v*/*v*) in 400 µL DMBT (nominal TEBU conc. of 0.3 µM or 1 µM) were incubated for 30 min, then 200 µL of samples were transferred into the sample block to measure the TEBU content. The total amount of TEBU added to the tubes was about 40 pmol at the nominal concentrations of 0.3 µM, and 131 pmol at 1 µM (see Figure 2). Data on TEBU recovery and sample preparation are found in Figure S5 and Figure S6 respectively.

We also assumed that the ratio of ‘plastic TEBU’ to ‘medium TEBU’ (with or without cells) would be reduced by the addition of albumin to the medium (see Figure 2 and Figure S4). Therefore, we started a new experimental series with BSA-containing medium (DMB). We measured how much TEBU (1.0 µM nominal) remained in the soluble fraction (medium + cells) when we varied the number of suspended cells (from 0.3% of total volume up to 3%). The TEBU recovery was 60–70%, and did not differ significantly between the conditions (varying the cell amount) (Figure 5A and Figure S5h–j). These data confirmed that there was considerably less plastic loss in the medium with BSA (DMBT). It also showed that the number of cells had no significant additional effect (in the range of 0.3–3% cell content).

In a follow-up study, dose proportionality was tested using a 3% cell suspension in DMBT medium. The recovery of TEBU in the soluble fraction was proportional to the amount added (Figure 5B and Figure S5g,h). This data suggested that the setup was suitable to measure cellular fractions, provided that the cells were separated from the medium in an additional experimental step.

### 2.5. Measurement of TEBU Accumulation in Cells

**Figure 6:**
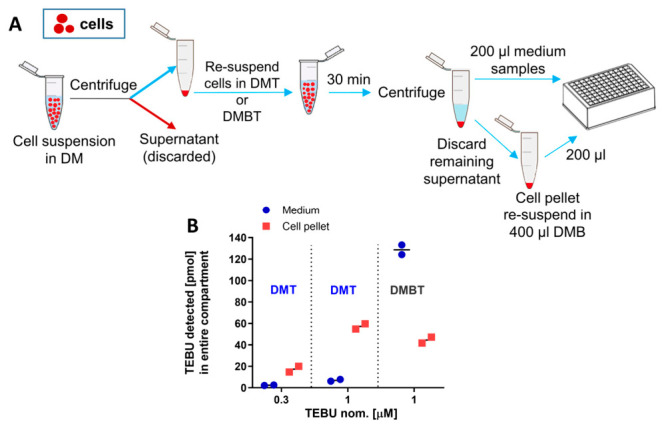
The distribution of TEBU between medium and cells. (**A**) A schematic of the experimental workflow. Proliferating LUHMES cells (3% *v*/*v* density) were suspended in 400 µL of DM, centrifuged, and the supernatant was discarded. Cell pellets were then resuspended in 400 µL of either DMBT or DMT. Note that the actual amounts of TEBU (in the DMT/DMBT added) were about 30, 100, 131 pmols (see Figure 2), as calculated from measurements of the master stocks (see Figure S3). After 30 min incubation, the samples were centrifuged to separate the medium and cells. An aliquot of the supernatant was used for LC-MS analysis. The remaining medium was discarded, and cell pellets were resuspended in 400 µL of DMB. From this suspension, an aliquot was used for analysis. (**B**) The TEBU contents in medium and cells are indicated. The conditions d, e, f from Figure S5 are shown here from left to right.

We modified the experimental protocol by including a centrifugation step to separate cells and medium. For calculations of potential cellular accumulation, the plastic-bound fraction was regarded as ‘fixed loss’. This is based on our experience that plastic-bound TEBU required solvents like acetonitrile or DMSO to be detached, while it was not affected by aqueous medium under normal laboratory conditions (Figure 6). Under these conditions, it was experimentally easy to separate cells from the medium, to determine the amount of compound in each fraction and to calculate the uptake ratio or a relative distribution ratio. We feel that this procedure would be difficult for adherent cultures (or sometimes even impossible), as there is always uncertainty about how much compound is bound to the plastic, relative to the amount bound inside the cell layer (also when cells are detached by trypsin or scraping).

For the cell uptake experiment (using suspension cultures), TEBU was used at two concentrations in DMT medium (0.3 and 1.0 µM nominal, no BSA) and at 1.0 µM in DMBT (added BSA). Measurements in all fractions gave reproducible and plausible results (Figure S5d–f): There was good dose proportionality in the DMT condition; the recovery was much higher (130%) in DMBT than in DMT (64%). The lower recovery in DMT is most likely a result of more plastic binding. Of the amount of TEBU in cell suspensions, most (>85%) was within cells in DMT medium (Figure S7). In contrast to this, a large fraction of TEBU (74%) was outside cells in DMBT medium. This finding may be explained by the fact that in DMT, TEBU preferentially binds to lipids (or some proteins) within cells, while in DMBT, it binds to the high amount of BSA in the medium.

**Figure 7:**
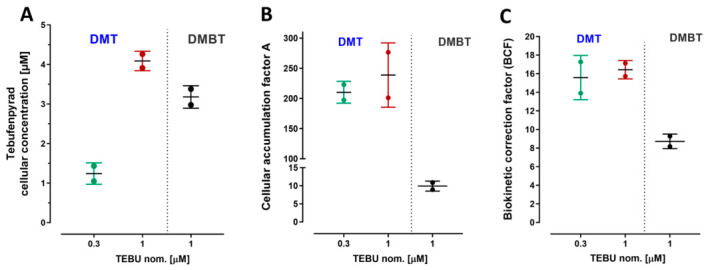
The estimated cellular concentrations and accumulation behavior of TEBU in LUHMES cells. Cells were exposed to DMT containing TEBU nominal concentration of 0.3 µM and 1 µM, and to TEBU in DMBT with a nominal concentration of 1 µM. (**A**) The cellular TEBU concentrations were calculated using the amount of TEBU detected in the cell pellet (see Figure S7), assuming that 10 million LUHMES cells have a total volume of 14 µL. (**B**) The cellular accumulation factor (CAF) was calculated as the ‘ratio of the measured cell and medium concentrations’. (**C**) The biokinetic correction factor (BCF) expresses the ‘ratio of the measured cell concentration, relative to the actual concentration of TEBU present in the medium added to the cells at the start of the experiment’ (see Figure S7). The conditions in the data displays (from left to the right) correspond to d (green), e (red), f (black) in Figure S5.

On the basis of data obtained for the cell fraction and cell volume (3% *v*/*v* cells in medium), the cellular concentrations were calculated. They were about 4 µM in DMT medium and 3.2 µM in DMBT medium (Figure 7A and Figure S7). Thus, the total cellular concentrations of TEBU were in the same range in DMT vs. DMBT medium (for same nominal concentrations), although the actual concentrations in medium differed about 20-fold (Figure S7).

### 2.6. Calculation of Distribution Measures

Using the data on the actual (measured) concentration in the medium, we calculated the ratio of medium vs. cell concentrations of TEBU. We named this ratio the ‘cellular accumulation factor (CAF)’, as this ratio gives an indication of the measured difference in total concentrations in medium and cells. It needs to be considered a descriptor of test compound behavior in a given experimental system, and it is strongly dependent on many experimental variables. In DMT, the CAF was in the range of 210–250 (Figure 7B, for 0.3 µM and 1.0 µM nominal concentrations). The consistency of the factor across the given concentration range is due to the dose-proportional effects observed throughout our experiments (Figure 7A, Figures S5d,e and S7). The CAF in DMBT was approximately 10 (Figure 7B). Thus, the total concentrations of TEBU inside and outside cells showed a much smaller gradient in the BSA-containing medium (due to strong protein binding in the medium).

Another perspective on describing the data of the cell accumulation experiments is highlighting the difference between the concentration of test compound (TEBU) added to the cell culture setup (Figure S7), and the concentration measured within cells. Such a measure may be used as indication on how far a PoD, based on nominal concentrations, may differ from the PoD, based on actual cellular concentrations [55]. This ratio may be considered a biokinetic correction factor (BCF). We calculated such measures here as A/B, with A being the measured cell concentration, and B being the actual concentration of TEBU present in the medium at the start of the experiment. The BCF was in the range of 16 for DMT medium and in the range of 10 for DMBT medium (Figure 7C). This suggests (for the DMBT setup) that the total concentrations of TEBU in cells were 10-fold higher than what was actually present in the original incubation medium. Notably, this refers to average cellular concentrations (not considering differences between compartments) and it applies to the total concentration (not the unbound fraction). To obtain more detailed data, highly sophisticated analytics are required. An alternative is computational modeling.

### 2.7. Prediction of TEBU Partitioning Using VIVD

In parallel to the bioanalytical assays, we used the VIVD [4] in silico prediction model to generate data on the likely TEBU partitioning to the cells and within cells. The model used data on (i) physicochemical properties of TEBU, (ii) the test systems’ characteristics (LUHMES cell suspensions), and (iii) the experimental conditions (e.g., medium volume, protein and lipid constituents of the medium, and vessel shape/dimensions). To account for plastic binding in the VIVD model, the plastic–medium contact surface area was calculated based on the container dimensions (Figure S8).

**Figure 8:**
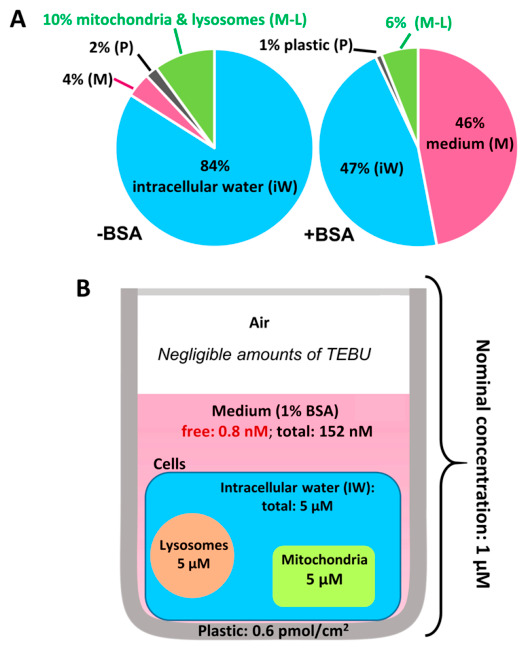
Evaluation of VIVD model predictions for TEBU distribution across assay compartments under different experimental conditions. The VIVD model was used to predict partitioning of TEBU, based on its physicochemical properties and the composition of the experimental system (cells, medium, plastic surface). Detailed data are found in (Figure S9). (**A**) The relative distribution (based on the number of molecules per fraction) was plotted for medium (M), intracellular water (IW), plastic (P), and combined mitochondria-lysosomes (M-L), assuming that the total TEBU input was 400 µL of a 330 nM solution (131 pmoles in total) in BSA-containing (DMBT) medium, and 400 µL of a 250 nM solution (100 pmoles in total) in BSA-free (DMT) medium (the actual measured concentrations of input were used here; nominally both media contained 1 µM TEBU (see Figure S5). Percentages in the figure represent the fraction of TEBU in each compartment relative to the total amount added to the system. (**B**) A schematic illustration of the distribution of TEBU in a cell culture well for the condition with BSA in the medium (condition f in Figure S5), according to the in silico biokinetics prediction model. Data for the medium without BSA are displayed in Figure S10A. A comparison of predicted and experimental data is provided in Figure S10B.

For experiments performed in DMT (1 µM nominal) the cellular concentration was predicted to be 7 µM (note: 4 µM was measured). The medium concentration prediction was 9 nM (17 nM was measured). Thus, the modeled data were in good agreement (≤2-fold offset) with the measured data (also for the 0.3 µM nominal concentration) (Figures S9 and S10A). For the experiments performed in DMBT (1 µM nominal) the cellular concentration was predicted to be 5 µM (note: 3.2 µM was measured). The medium concentration prediction was 152 nM (300 nM was measured). Thus, here also the modeled data were in good agreement (≤2-fold offset) with the measured data (also for the 0.3 µM nominal concentration) (Figure 8B and Figure S9). The information on various cell compartments allowed for a visualization of differential TEBU distributions in the presence or absence of BSA (Figure 8 and Figure S10A).

The prediction (P) to experimental (E) ratios (P/E) for the medium were 0.47–0.51 for all three conditions examined, and the P/E ratios for the amount of TEBU in cells were 1.59–1.67 for all conditions (Figure S10B). Thus, the model slightly over-predicted cell accumulation. However, all prediction accuracies (P/E ratios) were still within a generally accepted range of a 2-fold variation. Moreover, the VIVD model provided data not easily accessible by experimental approaches (subcellular distribution and unbound concentrations).

### 2.8. Consideration of Total vs. Unbound Concentrations

The VIVD model calculates the unbound fraction (f_u_) of the test compound, based on its logP and on the composition of the test system. This data can be used to calculate the unbound concentration (Cu) by multiplying the total concentration by f_u_. Cu is a measure for the concentration (and amount) of TEBU that is free to interact with toxicological targets and that determines diffusion processes, e.g., across cell membranes. It is important to note here that according to the free drug theory [56,57], the unbound concentration of the test compound is the same in medium and in cells (for neutral compounds, such as TEBU). A particularly notable finding from the VIVD model is that the free TEBU medium concentrations in DMT and DMBT were 1 nM and 0.8 nM (Figure S9), respectively. They hardly differed from one another, but they were much lower than their respective VIVD-predicted total medium concentrations (9 nM and 152 nM in DMT and DMBT, respectively). The free (low nM) concentrations did not only apply to the medium, but also to the cells (see free drug theory above). They differed by > 3 orders of magnitude from the total cellular concentrations (5–7 µM) (Figure 8B and Figure S10A).

The large differences between concentration measures (e.g., Cu, total concentration, nominal concentration, etc.) have major toxicological implications: which one should be used to extrapolate test method data to humans? In theory, the Cu should be a favored measure, at least where targets are involved that are freely accessible and solubilized in a ‘watery’ environment. Earlier work on compounds targeting the estrogen receptor (soluble protein in the nucleo-cytosolic compartment) has provided data in support of this suggestion [6]. However, the target of TEBU (cI of the mitochondrial respiratory chain) is not freely accessible (localization in the inner mitochondrial membrane), and it is embedded in a lipid-rich environment (membrane protein). Under such conditions, the local concentrations are hard to predict, and possibly the amount of compound per cell also plays a role for local intramembrane concentrations.

One indirect approach to address this issue is to make use of the strong modulatory effect of albumin on medium concentrations. If the free concentration (similar in the presence or absence of BSA) was the key determinant of toxicity, then BSA in the medium should not affect the apparent toxic potency of TEBU. If the total medium concentration plays a role, toxicity may be attenuated by albumin.

### 2.9. Consequences of Medium Protein Content on Toxicity Endpoints

**Figure 9:**
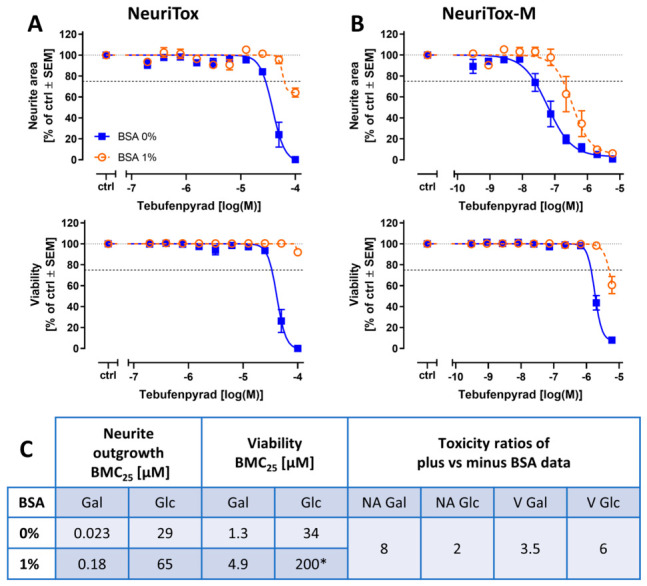
The influence of BSA in the test medium on TEBU-induced neurotoxicity. LUHMES cells were differentiated for 48 h and then treated for 24 h with TEBU under two different assay conditions: NeuriTox and NeuriTox-M. In both assays, the treatments were carried out either in the absence or presence of 1% BSA in the assay medium to assess the impact of protein binding on test compound toxicity. After treatment, the cells were stained with calcein-AM and Hoechst H-33342, and imaged using the Cellomics CellInsight microscope (Thermo Fisher Scientific, Waltham, MA, USA). The neurite area (NA) and viability (V) were quantified by automated image analysis and normalized to the DMSO (0.1%) control. Concentration–response curves show NA and cell V for the (**A**) NeuriTox and (**B**) NeuriTox-M assays. (**C**) A summary of the benchmark concentrations (BMC_25_ [µM]) for each test condition. The ratio of BMC_25_ (1% BSA)/(0% BSA) was calculated as an indicator of the effect of protein binding on TEBU toxicity. * When a BMC could not be determined, it was imputed.

Having observed the large consequences of medium protein content for test compound distribution in culture dishes (and into cells), we asked whether this would indeed affect the results of toxicity tests. In a simple setup to address this question, we performed toxicity testing in the NeuriTox and NeuriTox-M assay in the presence or absence of 1% BSA. In the NeuriTox assay, the neurite toxicity threshold concentration of TEBU was reduced by BSA by 50%. More pronounced effects (a 6-fold reduction) were observed for the cell viability (Figure 9A). In the NeuriTox-M assay, the sensitivity shift effected by BSA was 4–8-fold (Figure 9B). Notably, the BSA concentration used here was not excessive; it corresponded to about one fifth of what is commonly found in plasma. Similar data were observed, when we measured an endpoint directly related to the intracellular target of TEBU, i.e., the mitochondrial respiration. BSA caused a 4-fold loss of ‘apparent potency’ of TEBU (Figure S11). Thus, potency data from NAM depend on the exposure conditions (type of medium).

In another set of experiments, we checked whether human serum albumin (HSA) had similar effects as BSA. The data fully confirmed the earlier observations, as HSA reduced the apparent toxic potency of TEBU by 4–7-fold (Figure S12).

In the next step, we checked whether the need to be careful about experimental conditions would apply to all mitochondrial toxicants. We chose MPP^+^ as an alternative test compound, as it has the same target as TEBU (cI), but is a charged hydrophilic compound. Indeed, the toxicity of MPP^+^ was neither affected by BSA (Figure S13) nor by HSA (Figure S14). Thus, it appears that some compounds do not show the behavior observed for TEBU. This appears plausible, as both the protein binding and cell accumulation are strongly affected by physicochemical properties [13]. The main parameter used to predict such behavior is the logP, which is ~4.93 for TEBU and only 2.7 for MPP^+^.

**Figure 10:**
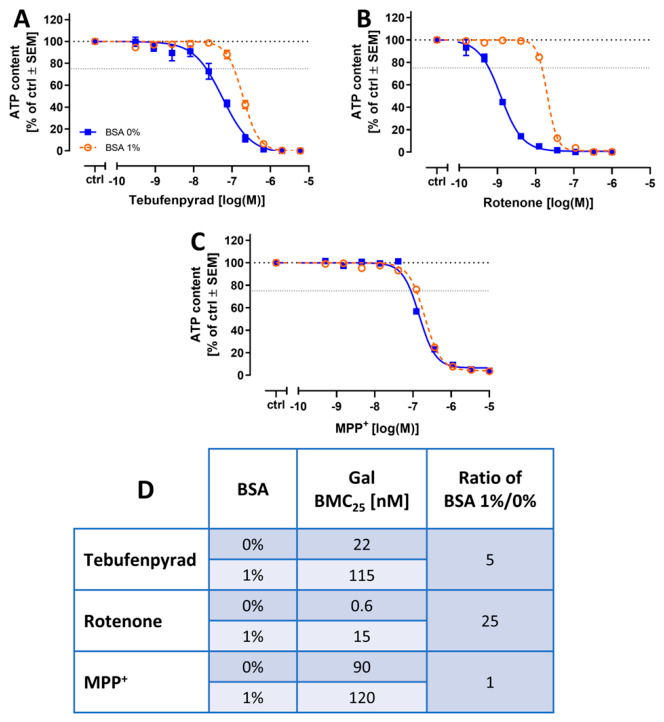
The effect of BSA on ATP loss induced by mitochondrial inhibitors in the NeuriTox-M assay. Concentration–response curves showing the ATP content after 24 h exposure to (**A**) TEBU, (**B**) rotenone, or (**C**) MPP^+^ in LUHMES cells cultured in galactose medium, with or without 1% BSA. Data are shown as relative to the DMSO (0.1%) solvent control (means ± SEM, *n* = 3). (**D**) A summary table of BMC_25_ values (concentration causing 25% ATP reduction) for each compound, with and without BSA. The ratios indicate fold changes in potency between 1% and 0% BSA conditions.

Next, we included rotenone in the comparison. This compound is one of the most frequently employed tool compounds for mitochondrial inhibition. It is a non-competitive inhibitor of cI in the single-digit nM range, and is similar to TEBU in this respect [58]. Rotenone is often used as a positive control for developmental neurotoxicity assays [59], and it is hydrophobic (logP = 4.1), like TEBU. We used the cellular ATP content as an easily accessible biochemical parameter of the toxicants’ cellular activity. In the NeuriTox-M assay, TEBU showed the expected shift in potency in the presence of BSA; MPP^+^ did not show such a shift (Figure 10). Both findings were as expected. When cells were exposed to rotenone, BSA caused a sensitivity shift of 25-fold (Figure 10). Thus, TEBU does not appear to be an extreme case amongst the hydrophobic compounds.

In a final set of experiments, we tested tolfenpyrad (logP of 5.6), another hydrophobic methylpyrazole, to see whether TEBU data were confirmed. Similar to TEBU, tolfenpyrad losses due to plastic binding were very high (Figure S4). In fact, the compound was so sticky that it could not be reproducibly handled in media, using normal laboratory equipment. The sticking to pipette tips made the normal dilution process of the compound in medium in predilution plates impossible. Concentration–response curves were established by serial predilutions of the compound in DMSO (where it does not stick), followed by a direct transfer of such stocks onto the cell cultures used for toxicity testing (no predilution plates). Based on the nominal concentrations used, BSA caused a 9-fold shift in the cytotoxicity threshold concentration (2.5-fold for the neurite area, 3-fold for ATP) in the NeuriTox-M assay (Figure S15). This largely confirms the finding obtained with TEBU.

## 3. Discussion

This study was initiated to exemplify issues that may arise when effect threshold concentrations (i.e., PoDs) are determined in NAMs, with the purpose of using them for predictions of threshold doses in vivo. IVIVEs might produce wrong results, if the nominal concentrations of test compounds largely differ from toxicity-relevant concentrations (e.g., cellular concentrations or free concentrations or target site concentrations) [18,60,61]. At present, there is no clear guidance on how to define the ‘relevant type of concentration’, and by which means to predict a potential offset between nominal concentrations and such measures. We have shown for one exemplary compound, TEBU, that free, nominal and cellular concentrations may differ by several orders of magnitude. We argued that this is more likely to happen for hydrophobic compounds than for chemicals with a low logP. We also suggest that the considerations are particularly important for compounds with intracellular targets. This limits the group of ‘problem compounds’.

Another feature may also contribute to the extent of problems that may be encountered: we chose here a potent toxicant with a defined cellular target for which interactions occur in the nM range. This means that low test compound concentrations were used in the assays. Under such conditions, binding to protein, lipid and plastic may have larger relative effects on remaining free toxicant concentrations than during testing at high concentrations. Only a small group of toxicants, mainly drugs and pesticides, have such high potencies. It will be important to identify these compounds and then to consider potential corrections of threshold concentrations by knowledge of their biokinetic behavior. The increased relevance of plastic binding at low concentrations has also been shown by others for certain drug classes [27]. However, there is a dearth of literature on whether plastic binding is saturable, under which conditions such saturation may occur, and whether this is affected by the coating of plastic with protein or cells. It is also unclear how far other distribution predictions (e.g., binding to different lipid classes and aggregate forms) is optimally captured by the SIVA model or other approaches [5] for all cell culture models. On the way to such knowledge, it appears important to perform various comparative studies that involve simultaneous measurements and modeling, and subsequent model refinement. Potentially, measurements also need to be refined, e.g., by using solid phase microextraction (SPME) [62] or rapid equilibrium dialysis (RED) to directly assess free concentrations. The use of optical/spectroscopic methods would allow us to determine subcellular localization of suitable model compounds with high resolution [63,64,65]. For very hydrophobic compounds (exemplified here by tolfenpyrad), it may be important to use an acoustic dispenser for test compound addition without contact to pipette tips or other plastic surfaces. Another (or additional) approach to reduce plastic binding may be to use glassware for the preparation of test compound solutions, and possibly for transfer and dispensing [66].

In our study, we found excellent predictivity of the VIVD model for the measured data (within factor 2) in medium and whole cells. The added benefit of the VIVD model was the information provided on free concentrations, and on subcellular concentrations. However, it needs to be noted that this data has not been confirmed experimentally here, and a measure of uncertainty is not implemented. In some studies, a sensitivity analysis has been used to map the extent of model uncertainty [5], but this requires knowledge of uncertainty of input parameters (e.g., logP of the test compound) to be applicable to defined use cases. The experimental effort to do so is quite significant: It may require a determination of the lipid profiles of the cells used for a given NAM, and possibly also of its cellular compartments. Moreover, the amounts of various test compounds in cellular organelles would need to be measured. Possibly one may even have to distinguish the organelle matrix from the surrounding membrane.

One of the more astonishing findings of this study was the extreme degree of plastic binding of methylpyrazoles. Our data suggest that the extent of compound loss may depend on the type of plastic (and possibly on its surface structure) [67]. This level of detail has not been factored into the VIVD model, and only a few data are available on this topic in the literature. With the increased use of NAMs, not just to study toxicity mechanisms or to determine relative toxicities, more extensive studies on in vitro distribution phenomena will be required. At present, this is a niche area, and there is no consensus on the best experimental setups. In this context, we also want to note that the information gained in this study is mostly based on 2–3 independent experiments. While this approach is common in cell biology, higher replicate numbers may be considered in regulatory toxicology studies, where data uncertainty should be kept as low as possible.

Another important outcome of our study was that cell accumulation data are likely to become more robust and sensitive, when the cell volume is high compared to the medium volume, and when the cell fraction is easy to distinguish from the plastic fraction. One solution for this is the use of cell suspensions (as done here). An advantage of this approach is also that the determination of cell volume is easier for suspended cells (with more or less spherical shapes), compared to adherent cells with a very complex shape. A potential disadvantage may be uncertainties introduced by the washing steps. Adherent cells may be washed in seconds to get rid of extracellular test compound in residual medium or loosely attached to the outer side of the cell membrane. For suspension cells, washing steps often require centrifugation, and they therefore take longer. This may favor a loss of intracellular compound to the wash medium. In our study, we tried to minimize such losses. In addition, we attempted to follow and document them by determining the mass balance of all fractions. In future studies this approach may be further refined and standardized.

An important conclusion from our study is that protein-free medium may increase uncertainties of biokinetics predictions. For many years, the field of NAM development has attempted to shift to serum-free media, ideally chemically defined, and often containing low amounts of protein, or no protein at all (as protein is usually extremely hard to standardize in purity). Our study shows that under such conditions, losses by plastic binding can be very high. Such conditions are nearly impossible to standardize across different laboratories. Moreover, extreme redistribution phenomena may occur between the cells and medium. In the presence of 1% albumin, we found such problematic effects to be largely attenuated. An interesting corollary finding was that the free TEBU concentration in albumin-rich and in protein-poor media differed only by about 20–25% (based on the VIVD model), and the total cellular concentration changed also only in this range. It appears that a lot of potential problems can be avoided by albumin addition, without largely changing the concentration of the test compound within cells.

It is of prime importance for toxicological testing whether the predictivity of the NAM gets better or worse by the addition of albumin to the medium. In this context, we found here that albumin increased the PoD of TEBU in the low single-digit (around 3–5-fold) range (when considering nominal concentrations). This is not negligible, but may be acceptable in relation to other uncertainties of NGRA [68]. The apparent loss in sensitivity is likely to be compensated by a gain of reliability, especially when comparing data from different laboratories (using different plastic ware and cell systems). However, this will need to be experimentally proven. It is also interesting to observe the potency shift while the cellular total concentrations and the free concentrations differed relatively little in experiments with and without albumin. The VIVD prediction of free concentrations would require experimental verification in the future. If confirmed, one can only speculate on the biochemical mechanisms responsible. One may assume that the concentrations leading to toxicity were roughly in the range of the affinity of TEBU for cI (low nM range). For an interaction according to the law of mass action, the effect of a concentration shift would be maximal at this concentration range: Assuming a Hill slope of 1, a concentration increase by a factor of 2 would change inhibition from 50% (possibly tolerated by cells) to 67% (possibly onset of toxicity). Moreover, it is likely that neurons have a functional cI reserve (respiratory spare capacity). TEBU would then first reduce the spare capacity, without altering the actual oxygen consumption rate of the cells. When the spare capacity is exhausted, very small increases in the cI inhibitor may then lead to strong reductions in respiration and thus measurable toxicity. Under such conditions, the concentration–effect curve may be considerably steeper than assumed by a standard law of mass action model, with small changes in free concentration having a relatively large biochemical impact.

While this mechanistic explanation remains at present an assumption, one may also consider the data from the perspective of what the concentrations would mean for threshold doses. It would require access to a physiology-based kinetic model to perform an IVIVE procedure [15,16]. One such model has been published for TEBU [69]. Using such a model, we calculated the respective oral intake that would lead to a free plasma (or cerebrospinal fluid) concentration of 1 nM. A model run under standard conditions suggested the cognate intake to be 0.01 mg/kg body weight per day (30 nmoles/kg/day). Incidentally, this is exactly the same value as the acceptable daily intake (ADI) recommended for TEBU. Although these data are preliminary and require further refinement, such ballpark estimates may provide a rough delineation of relevant doses and concentrations. The approach indicates how biokinetics data, as produced by our study, may be used in NGRA approaches. Much more detailed modeling, model parametrization, exploration of relevant model data, and a quantitative association to toxicity testing data is presently ongoing and will in the future complement and extend the information given here.

## 4. Materials and Methods

### 4.1. LUHMES Cell Culture

LUHMES cells are a sub-clone of a neural precursor cell line, which was derived from ventral mesencephalic tissue of an 8-week-old female human embryo [30]. The cells, with ATCC number CRL-2927, have a normal karyotype and primary gene sequence. They are modified by a conditionally active v-myc transgene with a known insertion site and control by a tet-on system. They have been short tandem repeat (STR)-authenticated [31].

LUHMES cells were cultivated as described previously [7,70]. In brief, the cells were grown in standard cell culture flasks pre-coated with 50 μg/mL poly-L-ornithine (PLO; Sigma, Steinheim, Germany) and 1 μg/mL fibronectin (Sigma, Steinheim, Germany). The maintenance culture was kept in proliferation medium (PM) consisting of advanced DMEM/F12 with 2 mM L-glutamine, 1 × N2 supplement, and 40 ng/mL fibroblast growth factor (FGF-2; Bio-Techne, Minneapolis, MN, USA). For differentiation, the medium was changed to differentiation medium (DM) consisting of advanced DMEM/F12 supplemented with 2 mM L-glutamine, 1 mM dibutyryl cyclic adenosine monophosphate (dBcAMP; Sigma, Steinheim, Germany), 1 μg/mL tetracycline (Sigma, Steinheim, Germany), and 2 ng/mL Glial Cell Line-Derived Neurotrophic Factor (GDNF; (Bio-Techne, Minneapolis, MN, USA) on day of differentiation 0 (d0).

### 4.2. NeuriTox Assay (UKN4a Test)

A previously published detailed protocol [7,44,71] was used. Cells were at passage #18–20. Briefly, the cells were seeded into coated 96-well plates at a density of 100,000 cells/cm^2^ in DM. Then the cells were treated with compounds one hour after the attachment. The exposure lasted for 24 h, when cytotoxicity was assessed by high content imaging. Thirty min before imaging, 533 nM calcein-AM and 1 μg/mL H-33342 (both from Sigma, Steinheim, Germany) were added to the wells. Fluorescent imaging data were automatically obtained by a Cellomics ArrayScan VTI imaging microscope and analyzed by Cellomics Scan software, version 7.6.2.4-100x (Thermo Fisher, Pittsburgh, PA, USA) and all data processing was done by a validated algorithm [45,72,73]. The benchmark concentration (BMC) for a 25% effect size (BMC_25_) was calculated as described earlier [70]. When the BMC_25_ could not be directly determined (<25% toxicity within the tested concentration range), a value was imputed. When a BMC_10_ was available, the highest tested concentration was used; when no effect was observed at any concentration, twice the highest tested concentration was imputed.

### 4.3. NeuriTox-M Assay Modification (UKN4b Test)

The NeuriTox-M variant of the NeuriTox assay was used as detailed earlier [32]. Briefly, glucose in the differentiation medium was substituted with the same concentration of galactose on day 2 (d2). All other procedures remained the same as for UKN4a. The exchange of media sugars was designed to change cell metabolism (M), as illustrated earlier. Thus, NeuriTox-M is a variant of the NeuriTox assay with a predominant mitochondrial metabolism (M).

### 4.4. Seahorse Mitochondrial Stress Test

The overall mitochondrial respiratory function of live cells [74] was assessed using the MitoStressLUHMES (MSL) assay, as described by Delp et al. [32]. The cells were plated into coated Seahorse XFe24 plates at 100,000 cells/well on d2 of differentiation. One hour prior to analysis, the medium was replaced with Seahorse XF base medium, supplemented with 2 mM glutamine and 1 mM pyruvate and either 18 mM glucose or 18 mM galactose. Mitochondrial oxygen consumption was assessed upon the injection of compounds (or solvent). A sequential addition of 1 μM oligomycin, 1.5 μM carbonyl cyanide-4 (trifluoromethoxy)phenylhydrazone (FCCP) and 0.5 μM rotenone plus 0.5 μM antimycin A was performed to obtain data on different mitochondrial respiration states. Mitochondrial parameters were calculated after the normalization of oxygen consumption relative to the total cell numbers [75].

### 4.5. ATP Quantification

To measure intracellular ATP levels, the same assay plates, as used for the imaging readout, were used. Following image analysis, cells were lysed in-plate, and then ATP was measured luminometrically [75,76], using a commercial reagent mix (Promega CellTiterGlo 2.0), containing luciferase. The data were normalized to DMSO solvent controls.

### 4.6. Preparation of Reference and Sample Stock Solutions for TEBU Biokinetics Experiments

A 100 mM reference solution of TEBU was prepared by dissolving 33.3 mg of TEBU powder in 1 mL of DMSO in a 1.5 mL polypropylene Eppendorf microfuge tube. Aliquots (10 µL) in 0.5 mL tubes (polypropylene) were prepared and stored at −80 °C. To prepare a 1 mM sample stock solution, aliquots were transferred from −80 °C to −20 °C one day prior to use. On the day of the experiment, the stock was thawed and diluted 1:100 in DMSO, using 1.5 mL microfuge tubes.

### 4.7. Preparation of Experimental Solutions for Biokinetics Studies

All TEBU solutions that were used for experiments were diluted to the final (experimental) concentration directly into the respective experimental plastic ware. The medium used was DM. Sometimes, BSA was added to the medium (DMB). For media containing TEBU only, we used the acronym DMT, and for media containing both TEBU and 1% BSA, we used the acronym DMBT. The respective nominal TEBU concentration is always specified separately. The nominal concentration differed for most experiments from the measured concentration, as the stock solutions already showed losses (due to plastic binding). Overviews on this are given in supplementary figures, and calculations are based on measured data. Nevertheless, we used the nominal concentrations for labeling experimental conditions in figures. For every preparation, first DM (or DMB) was pipetted, and DMT (or DMBT) was added in a second step. For preparations of 1 µM TEBU samples, 3 parts of DMT (or DMBT) were added to 1 part of DM (or DMB). For 0.3 µM TEBU solutions, 1 part of DMT (or DMBT) was added to 3 parts of DM (or DMB). In the case of standard preparations directly within a polypropylene sample block (a container that allows collection, freezing, storage and shipping of an array of 96 samples, obtained from Greiner Bio-One GmbH, Frickenhausen, Germany), a final volume of 200 µL was prepared; in experimental tubes and cell culture plates, the final volume used was 400 µL.

### 4.8. Preparation of Working Stocks in 50 mL Plastic Tubes

To limit the adsorption of TEBU to plastic surfaces, 50 mL tubes (Falcon, Corning, NY, USA) were pre-exposed to TEBU to ‘coat’ the plastic surface. Working stocks (DMT and DMBT) were prepared by diluting the 1 mM sample stock in 30 mL DM or DMB to a final concentration of 1.33 µM TEBU. These solutions were added to 50 mL polypropylene tubes and left for 8 h, before they were discarded. This coating cycle was repeated twice. The third and final filling was retained (as working stock) and used for subsequent experiments (Figure S2).

### 4.9. Preparation and Analytical Validation of ‘Gold Standards’

To minimize any potential loss of TEBU, ‘gold standard’ solutions were prepared directly within the sample block, without intermediate use of any plastic vessels (Figure S3). These standards were made by diluting calibration solutions (10 µM and 3.3 µM TEBU in DMSO) with DM directly inside the sample block. A calibration curve was produced from the gold standards. It was based on the peak area ratio of TEBU, normalized to the internal standard (diclofenac), plotted on the *y*-axis and the concentration of standards on the *x*-axis. The curves demonstrated good linearity and were used to convert chromatographic signals into actual TEBU concentrations. For all subsequent calculations, it was assumed that the recovery of the gold standard was 100%. The resulting calibration curve was then used to quantify TEBU levels in all samples throughout the study. It was in particular used to evaluate TEBU losses in working stocks (due to plastic binding). Direct measurements of TEBU ‘working stocks’ were performed in parallel to the gold standard quantification. The working stocks (DMT or DMBT of 1.33 µM TEBU) were diluted with their respective media (DM and DMB) inside the sample block. As a negative control, DM or DMB (without TEBU) were added to the sample block, and they gave no TEBU signal. From several measurements of working stocks, their actual concentration (different from the nominal conc.) and the percentage loss to plastic was determined and used for all further calculations.

### 4.10. Preparation of Cell Suspension Samples

For the preparation of cell suspension samples, proliferating LUHMES cells were used. Their total cell volume [13,45,77] is 1.4 µL per 1 million cells (1.4 pl/cell). In order to generate suspension samples with different cell densities, defined cell numbers (i.e., 1.1, 3.3, and 10 million cells) suspended in DM were transferred to 1.5 mL test tubes (Eppendorf, Hamburg, Germany). After centrifugation for 4 min at 300× *g*, the supernatant was discarded. To generate the cell suspension densities of 0.3%, 1%, and 3% (*v*/*v*) cells/medium, cell pellets were first resuspended in 100 µL or 300 µL of DMB (DM containing 1% BSA) to keep stress to the cells to a minimum. To reach a final volume of 400 µL for all samples, 300 µL or 100 µL working stock, containing 1.33 µM TEBU (DMBT), was added to obtain end concentrations of 1 µM or 0.3 µM TEBU. After 30 min incubation, the cell suspensions were used in two ways (Figure S6): (i) either 200 µL were transferred directly to the sample block; or (ii) the cell suspension was centrifuged, then 200 µL of the supernatant/medium was transferred to the sample block. Then the remaining medium was discarded and 400 µL DMB were added to resuspend the cells; 200 µL of this suspension was transferred to the sample block.

### 4.11. Preparation of Samples to Evaluate the Recovery of TEBU from Adherent Cell Cultures

LUHMES cells were differentiated until day 2 (d2) and re-seeded at a density of 220,000 cells/cm^2^ (400,000 cells per well) into a 24-well polystyrene plate (Sarstedt, Germany). The cells were left at 37 °C, 5% CO_2_ for 1 h to attach to the plate [78]. Note, this step is similar to the NeuriTox/NeuriTox-M assays, but the cell number used was 2.5-fold higher, to increase the contribution of cell volume to overall culture volume. The initial cell medium (DM) was removed, and test media (400 µL) were prepared directly in the cell culture wells as described above, using DM and DMT solutions. After 30 min of incubation, the samples of supernatant medium (200 µL) were transferred to the sample block. The remaining 200 µL were discarded. Then, 200 µL acetonitrile were added to the cell layer in each cell culture well. After 30 min of gentle shaking, 200 µL of DMB was added to each well and half of this solution (200 µL) was transferred to the sample block for measurement. In parallel, reference samples were prepared using the same handling procedure, but with cell-free wells. For these reference samples, only a single concentration, i.e., 1 µM TEBU (nominal) was used as the experimental solution.

### 4.12. Sample Processing and LC-MS/MS Setup

All the samples were collected in a sample block (Greiner Bio-One GmbH, Frickenhausen, Germany), and they were processed simultaneously by a robot. A volume of 800 µL acetonitrile (containing internal standard) was added to the samples (200 µL). Then the samples were centrifuged at 20,000× *g* for 5 min to remove precipitated proteins, and supernatants were used for further analysis (injected into the liquid chromatography–tandem mass spectrometry (LC-MS/MS) system) [79].

Chromatography was performed on a Shimadzu system (composed of automatic sampler SIL-20AC and SIL-20ACXR and HPLC pump LC-20AD and LC-20ADXR; Shimadzu Corporation, Kyoto, Japan) fitted with a Kromasil C18 (50 × 3 mm, 5 µm beads) column (NOURYON, Bohus, Sweden). Purified water with 0.1% formic acid (FA) (A) and acetonitrile with 0.1% FA (B) were used to apply a gradient. The gradient conditions were as follows: from 0 to 3 min ramp from 5 to 95% B; hold for 0.7 min (until T = 2.7 min); ramp over 2.71 min to 5% B; hold for 1.29 min (until T = 5 min) to re-equilibrate the system. The flow rate was 0.8 mL/min.

The mass spectrometry (MS) equipment consisted of an AB Sciex API 4000 controlled by version 1.6.3 of the Analyst software [44]. Tuning was performed in electrospray positive ionization mode with solutions of 0.05 µg/mL TEBU in acetonitrile/H_2_O (50/50 (*v*/*v*) + 0.1% FA) at a flow rate of 10 μL/min. For both full scan MS and multiple reaction monitoring (MRM) MS/MS, the mass spectrometer source parameters were as follows: ion spray voltage 5.5 kV and source temperature 550 °C. The nebulization and desolvatation gas (air) pressure were 50 psi each. Declustering potential and collision energy were 70 and 37 eV, respectively. The curtain gas pressure was 25 psi. The collision gas (nitrogen) pressure was 4 psi. The dwell time was 50 msec. The most favorable multiple reaction monitoring (MRM) transition monitored for TEBU was 334.3 (*m*/*z*) to 145.2 (*m*/*z*) [80]. Also, the LOQ and LOD for TEBU was 0.1 nM and 0.03 nM respectively.

All the samples were shipped in randomized positions in the sample block and with coded IDs. Calibration samples were included for each sample block, and they were coded as well. Based on this, the calculation of TEBU amounts in the samples were performed after the analytical runs by an independent scientist, knowledgeable of the code. Only the samples in the linear range of the calibration curve and above the limit of quantification were quantified.

### 4.13. Mathematical Simulations of TEBU in Cell Cultures

The experimental conditions of the bioanalytical assays were subsequently reproduced in silico using the VIVD model [4], implemented within Certara’s ‘Simcyp In vitro Data Analysis’ (SIVA^®^) Toolkit. The VIVD model was used to simulate TEBU’s partitioning into the cellular compartments, medium and plastic within the cell suspension assays (Figure S5, columns d–f).

### 4.14. Input Parameters for VIVD Modeling

The VIVD model requires three categories of input parameters: (1) the physicochemical properties of the test compound; (2) the assay’s experimental conditions; and (3) the cell type characteristics.

TEBU’s (US EPA CompTox-ID: DTXSID0034223) physicochemical properties were obtained from the CompTox dashboard [36]: TEBU is a neutral (pKa is not required), lipophilic (experimental logP: ~4.93) and non-volatile compound (predicted Henry’s law constant of 1.61 × 10^−11^ atm·mol/m^3^). The model was parametrized with a medium volume of 400 μL and a medium lipid concentration of 2.9 mg/L. The medium protein content was taken as 0.4 mg/mL (0.04% = 6 µM) as the baseline protein content in DMT and 1.04% in DMBT (baseline of 0.04% plus the additional 1% BSA), according to the supplier specifications, and in line with previous studies [7]. A temperature of 37° C was assumed.

The physiological characteristics of LUHMES cells were incorporated into the VIVD model. Volumetric fractions, membrane potentials and intramembrane pH values were estimated using the Simcyp simulator’s tissue composition module, and were used to determine compartment-specific membrane permeability [81,82,83]. The LUHMES parameters used in the model were as follows: cell volume (1.4 pL per cell; 14 µL per 10 million, as used in a typical experiment) [13,77], membrane potential (MP) (=−70 mV), intracellular water (IW) (fraction = 0.7467; pH = 7.12), mitochondria (fraction = 0.1; pH = 8; MP = −120 mV), lysosomes (fraction = 0.01; pH = 4; MP = 10 mV), neutral lipids (fraction = 0.0562), neutral phospholipids (fraction = 0.0621), acidic phospholipids (cellular concentration = 0.44 mg/g of cells, which was assumed to be equivalent to mg/mL of cells).

Note that the above data have been corrected for the absence of extracellular water. The Simcyp’s default tissue composition data assume a brain extracellular water content of 9.2%. This component was excluded from the simulated cellular volume. Therefore, the relative volume fractions of the remaining intracellular constituents (i.e., neutral lipids, neutral phospholipids, and IW) were re-calculated from the tissue data. As a result, these fractions are greater in the in vitro simulations (without extracellular water) than the corresponding Simcyp-predicted fractions under in vivo conditions (which include extracellular water).

### 4.15. Data Processing, Display and Statistics

For the toxicity evaluation of compounds in the NeuriTox assays, all the data were normalized to vehicle controls. Curve fitting for presentation in figures employed a four-parameter Hill function constrained between 0 and 100%. Benchmark concentration (BMC) values were computed using dedicated software [84], with data pre-normalized as per the method described by Krebs [70,85]. If not mentioned otherwise, the values are expressed as means ± SEM. If not indicated otherwise, the experiments were performed at least three times (i.e., using three independent LUHMES differentiations), with at least three technical replicates per condition. Graphpad Prism (version 9; GraphPad Software, San Diego, CA, USA) was used for data analysis and the plotting of figures. 

## 5. Conclusions

Our study showed that nominal, free, and cellular concentrations may differ significantly in cell-based assays. In particular, plastic and protein binding alters the bioavailability of lipophilic compounds. In such cases, it can be necessary to generate experimental data on test compound distribution. Our data suggest that VIVD modeling can be a useful complement or sometimes alternative to this, and that it is likely to improve in vitro exposure interpretation. Practical conclusions were that cell suspensions provided a practical strategy for measuring cellular accumulation, and that albumin reduced compound losses. The use of glass vials and minimizing plastic contact at all steps is likely to attenuate problems.

## Data Availability

All data not disclosed directly in the manuscript and its supplements are being made freely available upon request.

## Supplementary Materials

The following supporting information can be downloaded at https://www.mdpi.com/article/10.3390/ijms27125547/s1.

## Abbreviations

The following abbreviations are used in this manuscript:

ATP	Adenosine triphosphate
BMC	Benchmark concentration
BMC_25_	Benchmark concentration 25%
BSA	Bovine serum albumin
conc.	Concentration
DM	Differentiation medium
DMB	Differentiation medium with 1% BSA
DMBT	Differentiation medium containing tebufenpyrad and BSA 1%
DMSO	Dimethyl sulfoxide
DMT	Differentiation medium containing tebufenpyrad
Gal	Galactose
Glc	Glucose
GS	Gold standard
IVIVE	In vitro to in vivo extrapolations
LogP	Octanol to water partition coefficient
LUHMES	Lund human mesencephalic
MPP^+^	1 methyl 4 phenylpyridinium
NA	Neurite area
NAMs	New approach methodologies
NGRA	Next-generation risk assessment
nom.	Nominal
OCR	Oxygen consumption rate
pKa	Acid dissociation constant
PM	Proliferation medium
PoD	Point-of-departure
SD	Standard deviation
SEM	Standard error of the mean
SIVA	Simcyp in vitro data analysis
TEBU	Tebufenpyrad
V	Viability
VIVD	Virtual in vitro distribution
WS	Working stock
