# Supplementary material for: Complex Distribution Phenomena and Plastic Binding of Test Chemicals in Cell Culture Experiments: Exemplification by Tebufenpyrad
Source: Int J Mol Sci. 2026 Jun 19;27(12):5547. doi: 10.3390/ijms27125547 (PMC13300673; doi:10.3390/ijms27125547)
Supplement: Supplementary file 1 [file ijms-27-05547-s001.zip › ijms-4329264-supplementary.pdf]

Supplementary information for

Complex Distribution Phenomena and Plastic Binding of Test Chemicals in Cell Culture Experiments: Exemplification by Tebufenpyrad

Mahshid Alimohammadi[1], Hiba Khalidi[2], Elias Zgheib[2], Anna-Katharina Holzer[1], Naja Bürgers[1], Céline Brochot[2], Patrik Lundquist[3], Viktoria Magel[1], Baiba Gukalova[4], Edgars Liepinsh[4], and Marcel Leist[1, 5]*

Table of Contents

Figure number	Page	Short title
Fig. S1	1	Inter-operator-reproducibility of the NeuriTox and NeuriTox-M assays
Fig. S2	2	Preparation of the working stocks in 50 ml plastic tubes
Fig. S3	3	Preparation of 'gold standard' and 'working stock' solutions within a sample block
Fig. S4	4	Confirmation of extensive compound losses by storage in plastic vessels
Fig. S5	6	Overview of experimental conditions, nominal concentrations (amount) and measured (calculated from measured data) concentrations of TEBU
Fig. S6	7	Preparation of cell suspension samples
Fig. S7	8	TEBU percentage in each fraction and cellular concentration in LUHMES cells under various exposure conditions
Fig. S8	9	Calculation of the medium contact surface area of Eppendorf tubes
Fig. S9	11	Tabular overview of VIVD-predicted quantities of TEBU
Fig. S10	12	Distribution of TEBU in cell culture without BSA and comparison of predicted vs. experimental data.
Fig. S11	13	Effect of BSA on TEBU-induced inhibition of mitochondrial respiration
Fig. S12	14	Effect of human serum albumin (HSA) on TEBU-induced neurotoxicity
Fig. S13	15	Effect of Bovine serum albumin (BAS) on MPP^+ induced neurotoxicity
Fig. S14	16	Effect of human serum albumin (HSA) on MPP^+ induced neurotoxicity
Fig. S15	17	Influence of BSA and treatment handling on tolfenpyrad- induced neurotoxicity in NeuriTox-M assays
	18	Supplementary references

Graphical abstract

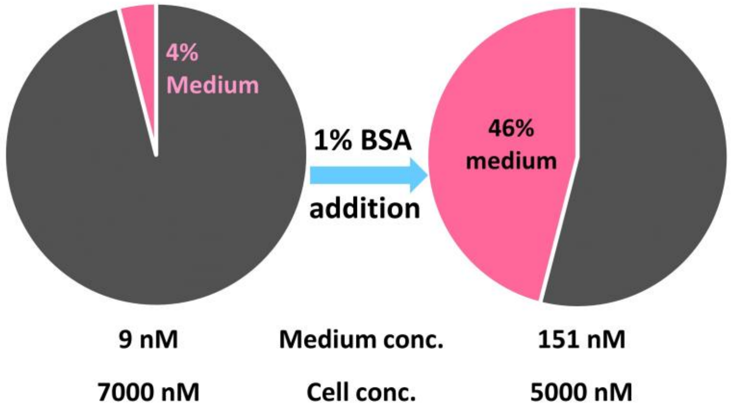

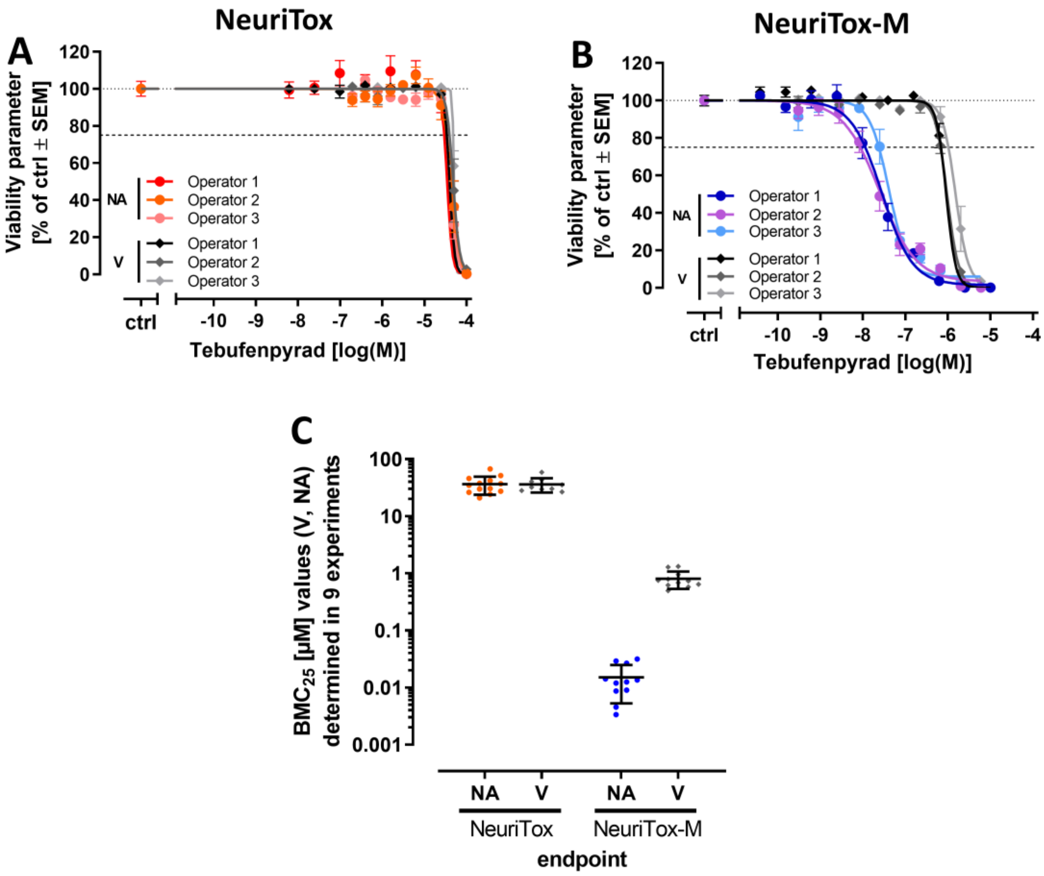

Supplementary Figure 1: Inter-operator-reproducibility of the NeuriTox (UKN4a) and NeuriTox-M (UKN4b) assays.

LUHMES cells were treated from d2 to d3 (for 24 h) with TEBU under (A) NeuriTox and (B) NeuriTox-M assay conditions, stained with calcein-AM and Hoechst H-33342, and imaged using the Cellomics CellInsight microscope. Neurite area (NA) and cell viability (V) were assessed using an automated algorithm and are shown relative to the DMSO (0.1%) solvent control (ctrl). (C) Data produced by three operators over several years are displayed in the graphs. NeuriTox data consist of 3, 8 and 3 individual experiments of operator 1, 2 and 3, respectively. NeuriTox-M data consist of 2, 6, and 3 individual experiments of operator 1, 2 and 3, respectively. Data are means ± SEM. Data generated by operator 2 corresponds to the data shown in Figure 1. This figure supports and provides additional detail for Figure 1 in the main text.

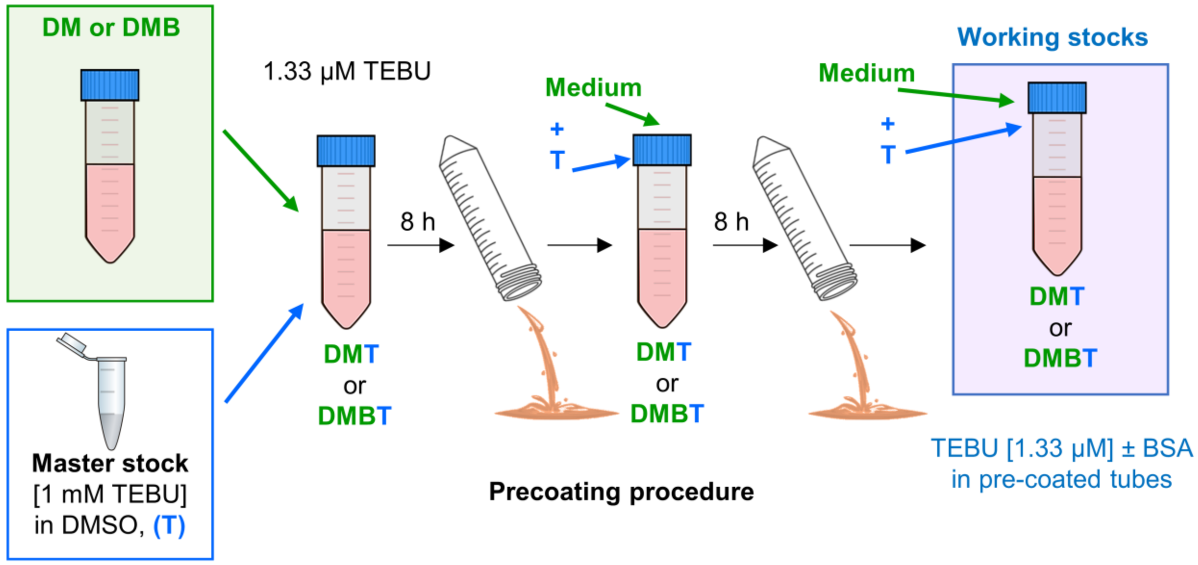

Supplementary Figure 2: Preparation of the working stocks in 50 ml plastic tubes.
Working stocks (DMT and DMBT) were prepared by diluting the 1 mM TEBU (in DMSO) master stock in medium (DM or DMB) to a final concentration of 1.33 µM (30 ml in total). These solutions were added to 50 ml polypropylene conical centrifuge tubes (Falcon, Corning, NY, USA) and left for 8 h, before they were discarded. Then the same solutions were generated again within the vessels used before. This procedure was repeated a third time, to pre-coat the plastic surface and reduce TEBU binding. The third and final filling was retained (as 'working stock') and used for subsequent experiments. DM: differentiation medium; DMB: differentiation medium with 1% BSA; BSA: bovine serum albumin; DMT: TEBU working stock in DM; DMBT: TEBU working stock in DMB. This figure supports and provides additional detail for Figure 2 in the main text.

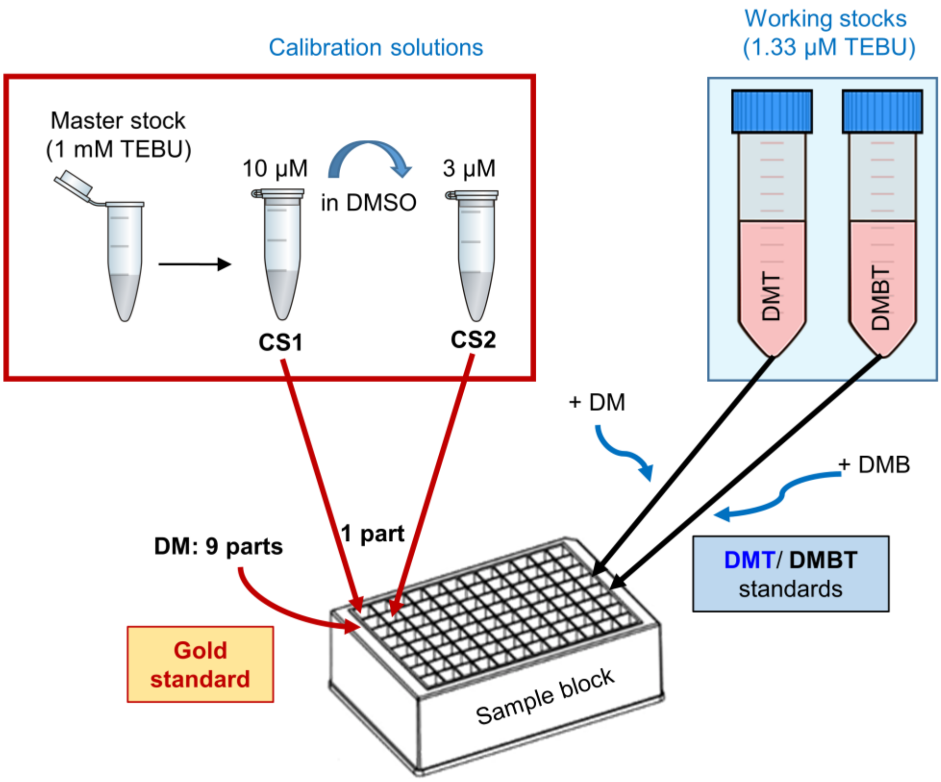

Supplementary Figure 3: Preparation of 'gold standard' and 'working stock' solutions within a sample block.

To produce gold standard calibration solutions (CS) of 10 µM (CS1) and 3 µM (CS2), TEBU-solutions were prepared in DMSO from the sample stock of 1 mM TEBU (in DMSO). Then, CS were diluted (1:10) with differentiation medium (DM) directly within the sample block (no further contact of plastic surface) to produce 0.3 and 1 µM 'gold standards'. To measure the TEBU concentration of working stocks (in either DMT or DMBT), solutions were used directly from the 50 ml tubes and diluted within the sample block with DM ± BSA to reach final concentrations of 1 µM (150 µL DMT + 50 µL DM) and 0.3 µM (50 µL DMT + 150 µL DM), or similar steps with DMB medium (*n* = 3). This figure supports and provides additional detail for Figure 2 and Figure 6 in the main text.

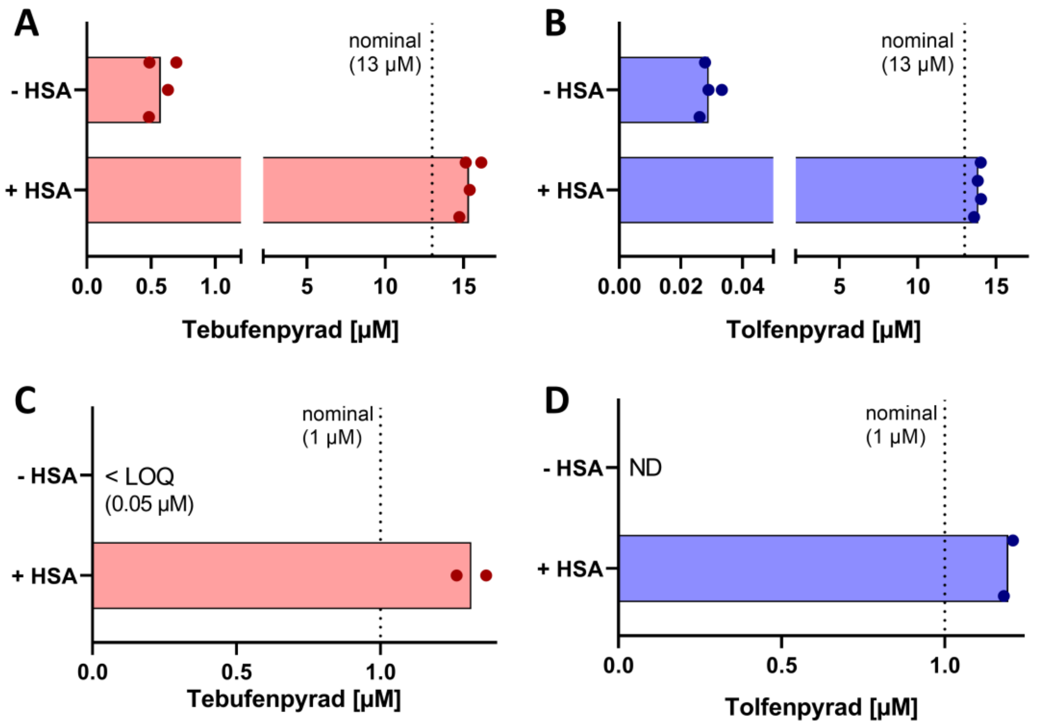

Supplementary Figure 4: Confirmation of extensive compound losses by storage in plastic vessels.

(A, B) Recovery of TEBU (A) and tolfenpyrad (B) from 50 ml centrifuge polypropylene tubes. Solutions were prepared as in Figure 1 within the tubes at a nominal concentration of 13 µM in medium without human serum albumin (-HSA) or medium supplemented with 0.5% human serum albumin (+HSA). Samples for analysis of compound "still in solution" were obtained after 5 h. Data points for all four experiments are shown; bars indicate the mean value. (C, D) Recovery of TEBU (C) and tolfenpyrad (D) from 1.5-ml microfuge polypropylene tubes. Solutions were prepared within the tubes at a nominal concentration of 1 µM in medium without human serum albumin (-HSA) or medium supplemented with 0.5% human serum albumin (+HSA). Samples for analysis of compound still in solution were obtained after 5 h. Data points for two experiments are shown; bars indicate the mean values.

The black dashed line indicates the assumed nominal concentration (either 13 µM or 1 µM). The nominal concentration represents the amount intended to be present in the sample theoretically. Samples of medium, without test compounds added (+HSA, -HSA) were also measured. They showed no detectable signal. LOQ = limit of quantification; ND = not detectable. This figure supports and provides additional detail for Figure 4 in the main text.

Methods for analysis of test compound recovery in Figure S4

Experimental procedure: TEBU and tolfenpyrad master stocks of 10 mM in DMSO were prepared. Two versions of blank medium were used: standard medium (DMEM/F12 with 1% GlutaMax, 1.55 mg/mL glucose, 0.1 mg/mL apotransferrin, 25 µg/mL insulin, 20 nM progesterone, 100 µM putrescine, and 30 nM selenium) and medium supplemented with 0.5% human serum albumin (HSA) (Sigma, Steinheim, Germany). For the experiments in 50 mL centrifuge tubes a base medium was prepared from the master stock and the blank medium,

resulting in a nominal concentration of 13 µM of TEBU or tolfenpyrad. For experiments in 1.5 ml microfuge tubes, media were prepared at an assumed nominal concentration of 1 µM by diluting the base medium (nominal 13 µM) with the respective blank medium. Samples were incubated for 5 h at room temperature before collection. All samples were stored at -80 °C until analysis.

Preparation of samples for chemical analysis: All samples were thawed and homogenized by shaking for 10 min at 100 rpm. A 50 µl aliquot of each sample was transferred to a liquid chromatography (LC) vial and mixed with 250 µl of acetonitrile: methanol (3:1, v/v) containing reserpine as an internal standard. The pipette tips were rinsed with the same solvent. The vials were vortexed thoroughly to achieve uniform mixing. When samples contained human serum albumin (HSA), the mixture was centrifuged, and the resulting supernatant was collected for liquid chromatography–tandem mass spectrometry (LC–MS/MS) analysis. Samples were either analyzed immediately or stored at -20 °C until LC–MS/MS injection.

Chemical analysis procedure: A "Waters Acquity LC system" coupled to a "Xevo TQ-S micro triple-quadrupole mass spectrometer" equipped with an electrospray ionization (ESI) source, operating in the positive ion mode, was used. Sample injections were carried out with a "Waters Acquity H-class autosampler". Chromatographic separation was achieved on a "Waters Acquity BEH C18 column" (2.1 × 50 mm, 1.7 µm particle size) maintained at 30 °C. The mobile phase consisted of 0.1% aqueous formic acid (phase A) and acetonitrile (phase B). The flow rate was 0.4 ml/min, and the injection volume was 1 µl. At the start of the run (0.0 min), the mobile phase consisted of 5% phase B and was held constant for 0.5 min. From 0.5 to 2.5 min, the proportion of phase B was gradually increased from 5% to 98%. The composition was then maintained at 98% phase B until 4.0 min to ensure complete elution of strongly retained components. After this period, at 4.3 min, the mobile phase was returned to 5% phase B, restoring the initial conditions. From 4.3 to 5.0 min, the system was re-equilibrated at 5% phase B in preparation for the next injection. The mass spectrometer was operated in multiple-reaction-monitoring (MRM) mode. The source parameters were optimized as follows: capillary voltage 3 kV, cone voltage 30 V, desolvation temperature 600 °C, cone gas flow 50 l/h, and desolvation gas flow 800 l/h. Nitrogen was used as both the nebulizing and desolvation gas. The cone and collision energies were tuned individually for each analyte to obtain maximum sensitivity. Detection and quantification were carried out for TEBU and tolfenpyrad, with reserpine used as the internal standard. Each technical sample was measured twice and averaged. The response was given as the ratio of the analyte peak area to the internal standard (resperine) peak area. The MRM transition for TEBU (m/z 334.10 → 117.00) was determined experimentally using the reference standard. Dwell time (0.10 s), cone voltage (20 V), and collision energy (35 eV) were optimized to maximize analytical sensitivity. For tolfenpyrad, the MRM transitions (m/z 384.10 → 117.00, 170.90, 197.00) were established based on published data in the absence of a reference standard[1,2]. The calibration reference for experiments in cell culture dishes, were prepared without plastic contact.

	Standard cell culture (no centrifugation)			Cell suspension (with centrifugation to separate cells and medium)			Cell suspension (no centrifugation and cell separation)			
**Sample ID**	**a**	**b**	**c**	**d**	**e**	**f**	**g**	**h**	**I**	**J**
**BSA%**	-			-	-	1	1	1		
**Applied working stock (WS)**	DMT			DMT	DMT	DMBT	DMBT	DMBT		
**TEBU nom. conc. [µM]**	0.3	1	1	0.3	1	1	0.3	1	1	1
**TEBU nom. amount [pmol]**	120	400	400	120	400	400	120	400	400	400
**Predicted TEBU amount in WS [pmol]**	30	100		30	100	131	40	131		
**Calculated loss in WS relative to nom. amount (%)**	75			75		67	67	67		
**Real TEBU conc. in WS [µM] calculated/measured**	0.075	0.25		0.075	0.25	0.33	0.1	0.33		
**Measured TEBU amount in entire compartment [pmol]**	17	62	58	19	64	172	25	80	78	95
**Real amount [%]**	57	62	58	63	65	131	62	61	59	73

**Container type**	24-well Sarstedt plate			1.5 mL Eppendorf tube			1.5 mL Eppendorf tube			
**Medium volume [µL]**	400			400			400			
**Cell number (million)**	0.4	0.4	-	10			10	10	3.325	1.1
**Plastic-medium contact surface* [mm²]**	232	232	269	278			278			
**Acetonitrile 200 [µL]**	Yes			No			N0			
**Incubation temperature**	37 °C			Room temperature						

Supplementary Figure 5: Overview of biokinetic experimental conditions (a–j), and the nominally applied and measured concentrations/amounts of TEBU in the in vitro assay compartments (plastic, medium and cells).

The figure summarizes, for each condition, the presence or absence of BSA, the applied working stock, nominal TEBU concentration and amount, and the measured TEBU concentrations and amounts (or values calculated from measured data) used for downstream analyses. All assays were conducted at a total volume of 400 µL. For TEBU quantification, 200 µL of the assay volume was transferred to the sample block, and reported amounts were normalized to the full 400 µL. When additional steps were performed to obtain a separate cellular fraction (cell lysis with acetonitrile or centrifugation followed by resuspension), an additional 200 µL of lysed (acetonitrile-treated) or resuspended cells was also transferred to the sample block. The table further reports relevant assay parameters, including container type, medium volume, cell number, plastic–medium contact surface area, and use of acetonitrile. Mass balance was calculated by summing TEBU amounts across all experimental compartments (e.g., plastic, medium, and cells) and relating this total to the measured amount of TEBU added to each experiment; values are reported as percentages. This figure provides supporting information for Figure 3, 5, 6, and 8 in the main text. The incubation time for all samples was 30 minutes.

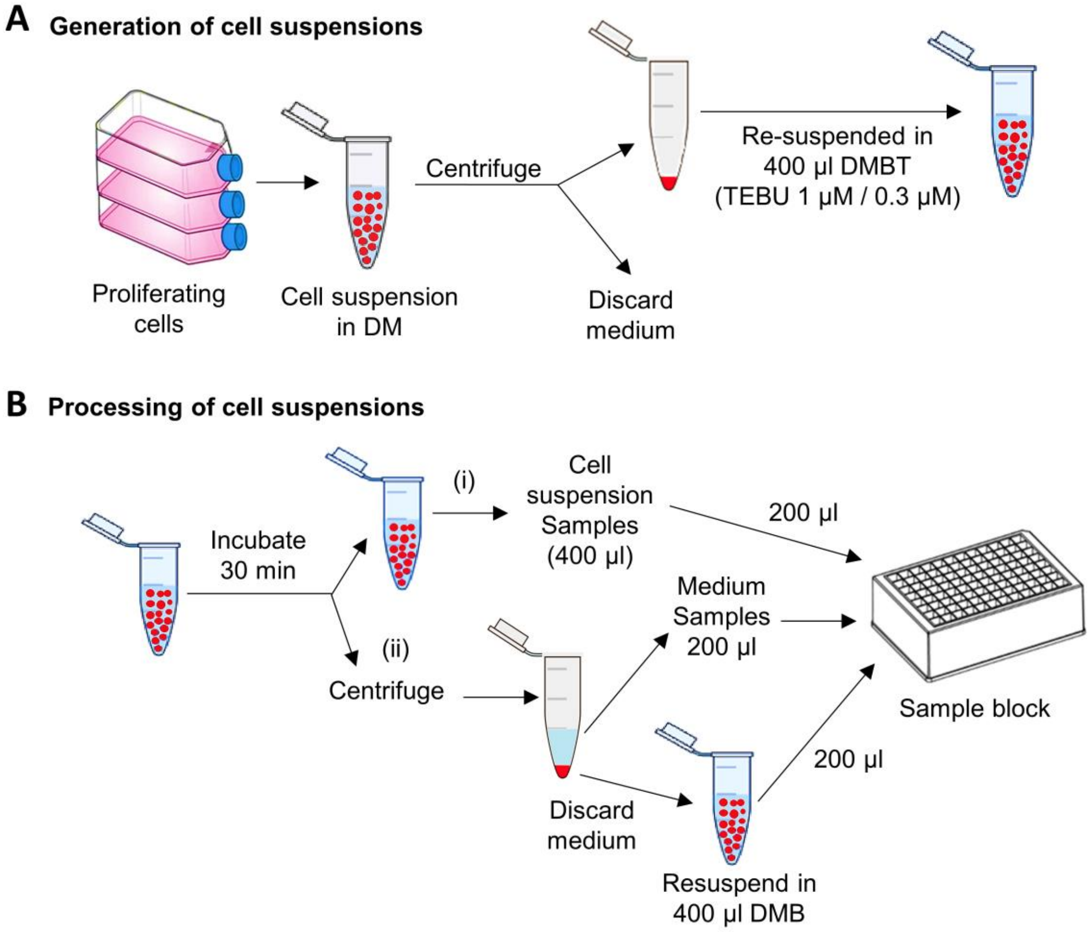

Supplementary Figure 6: Preparation of cell suspension samples.

(A) Cell suspensions of proliferating LUHMES cells with different cell densities were prepared. Then, samples were centrifuged. After removing the supernatant, cells were re-suspended in 400 µL of medium with TEBU (0.3 or 1 µM). (B) After 30 minutes of incubation, cell suspensions were used in two ways: (i) either 200 µL of the cell suspension was directly transferred to the sample block (upper path), or (ii) cells were separated from their medium. Medium was transferred to the sample block (the remaining supernatant was discarded). The cell pellet was re-suspended in 400 µL DMB. Then, 200 µL of the cell suspension were transferred to the sample block (lower path). This figure supports and provides additional detail for Figure 5 in the main text.

Working stocks nominal conc. [µM]	DMT [0.3 µM]	DMT [1 µM]	DMBT [1 µM]
TEBU, each compelete circle is 10 pmol<br>Cells, 10 milllion (14 µl)<br>DMT (400 µl)<br>DMBT (400 µl)	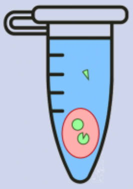	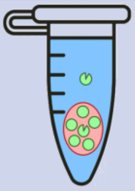	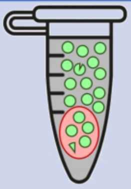
Nominal TEBU amount in the experiment	(120)	(400)	(400)
Measured TEBU amount [pmol] in the experiment	30	100	131
Measured TEBU amount in medium [pmol]	**2**	**7**	**128**
Measured TEBU amount in cells [pmol]	17	57	44
Total recovery amount in medium + cells [pmol]	19	64	172
Mass balance	19 of 30 (57%)	64 of 100 (65%)	172 of 131 (131%)
TEBU conc. added [nM]	75	250	330
Calculated TEBU conc. in medium [nM]	6	17	300
Calculated TEBU conc. in cells. [µM]	1.2	4	3.2
Fraction of TEBU in medium [%]	11	11	74
Fraction of TEBU in cells [%]	89	89	26

Supplementary Figure 7: TEBU percentage in each fraction and cellular concentration in LUHMES cells under various exposure conditions.

Proliferating LUHMES cells (3% v/v density) were suspended in 400 µL of either DMBT containing 131 pmol TEBU (nominal TEBU conc. of 1 µM) or DMT containing 33 or 100 pmol (nominal TEBU conc. of 0.3 µM or 1 µM respectively). After 30 min incubation, samples were centrifuged to separate medium and cells. An aliquot of the medium was used for LC-MS analysis. The remaining medium was discarded, and cell pellets were resuspended in 400 µL DMB. From this suspension, an aliquot was used for evaluating the TEBU content in the cells. In the figure a visual summary of TEBU distribution between medium and cell pellet is provided. Each full green circle represents 10 pmol of TEBU; partial circles represent fractions (e.g., 2.5 pmol for a quarter circle). The cell pellet corresponds to 10 million LUHMES cells (~14 µL volume) in 400 µL medium. This figure supports and provides additional detail for Figure 7 in the main text. Data correspond to the conditions d-f in Figure S5.

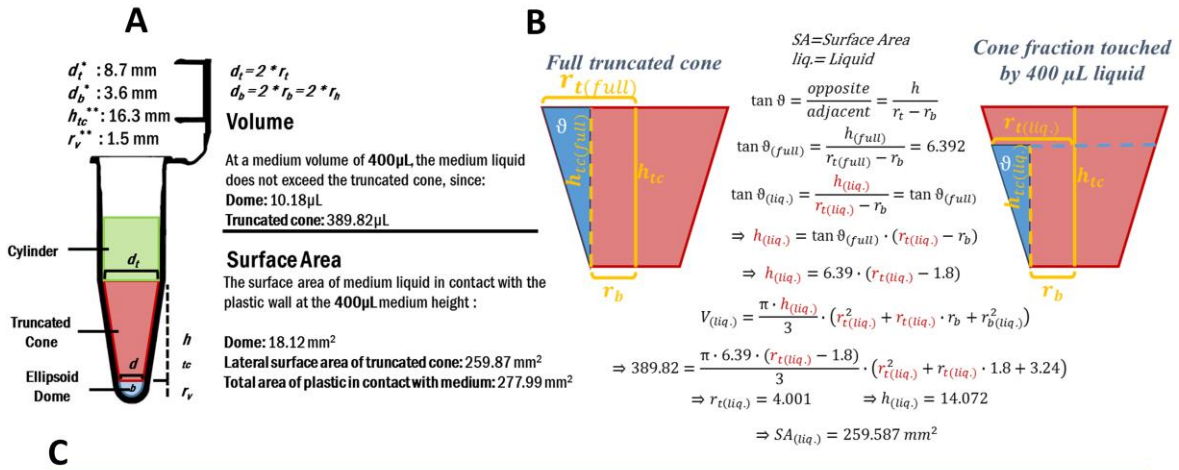

C

	Compartment	Volume	Lateral SA
1	Ellipsoid dome	$\frac{2}{3} \cdot \pi \cdot r_h^2 \cdot r_v$	$2 \cdot \pi \cdot \sqrt[p]{\frac{2 \cdot (r_h \cdot r_v)^p + (r_h^2)^p}{3}}$, where p = 1.608
2	Truncated cone	$\frac{1}{3} \cdot \pi \cdot h_{tc} \cdot (r_t^2 + r_t r_b + r_b^2)$	$\pi \cdot (r_t + r_b) \cdot \sqrt{(r_t - r_b)^2 + h_{tc}{}^2}$

Supplementary Figure 8: Calculation of the medium contact surface area of Eppendorf tubes.

(A) The geometrical representation of an Eppendorf tube comprises three regions: an oblate hemispheroid (blue), a truncated cone (red) and a cylinder (green). In the cell suspension assays, 400 µL of medium was added to the tube. At this volume, the liquid level remained in the truncated cone (red) and did not reach the cylindrical section (green), as the combined capacity of both the ellipsoid dome and truncated cone is 522.13 µL. Abbreviations: db, bottom diameter; dt, top diameter; htc, height of the truncated cone; rb, bottom radius of the truncated cone; rh, horizontal radius of the oblate hemispheroid; rt, top radius of the truncated cone; rv, vertical radius of the oblate hemispheroid. *Data extracted from manufacturer's datasheet, **Data estimated from digitised datasheet.

(B) Geometrical method for calculating the liquid–plastic contact surface area (SA) in a truncated cone. Left: schematic representation of the complete truncated cone, defined by bottom radius (rb), top radius of the full cone (rt (full)), and height of the full cone ($h$tc (full)). Right: schematic of the truncated cone partially filled with liquid, characterized by bottom radius (rb), the liquid height ($h$tc (liq.)), and the corresponding reduced top radius (rt(liq.)). In both cases, the vertical segment h is transposed from the central axis to the point of intersection between rb and the lateral surface, preserving its length, thereby facilitating the determination of the tangent of angle ϑ. Center: equations relating the geometry of the full and partially filled truncated cones, based on the conserved angle ϑ (and its tangent) between the liquid–air interface and the lateral surface. Abbreviations: htc, truncated cone height; htc(full), height of the full truncated cone; htc(liq.), height of the liquid-filled portion of the truncated cone; rb, bottom radius; rt(full), top radius of the full truncated cone; rt(liq.), top radius of the liquid-filled portion of the truncated cone; SA(liq.), liquid–plastic contact surface area; ϑ, angle between the liquid–air interface and the lateral surface of the truncated cone; tanϑ (full),

tangent of ϑ for the full truncated cone; tanϑ (liq.), tangent of ϑ for the liquid-filled portion of the truncated cone; V(liq.), liquid volume.

(C) Equations used to calculate the contact area between the medium and the plastic in 1.5mL Eppendorf tube. The ellipsoid dome SA equation was defined by the 'Matematiske Formler' book (Thomsen 1901) while the others were listed in the 33rd edition of the 'CRC Standard Mathematical Tables and Formulas' manual (Zwillinger 2018). Abbreviations: htc, height of the truncated cone; rb, bottom radius of the truncated cone; rh, horizontal radius of the oblate hemispheroid; rt, top radius of the truncated cone; rv, vertical radius of the oblate hemispheroid.

**A**	**With 1% BSA in medium (DMBT)**			
**compartments**	**Volume [µL]**	**VIVD predicted amount [pmol]**	**VIVD predicted molarity [nM]**	**predicted conc./WS conc.**
**Medium**	400	60	152	0.5
**$Medium_u$**	400	0.3	0.8	0.002
**$Cells_t$**	14	70	5000	15
**$Cells_M$**	1.4	7	5000	15
**$Cells_{IW}$**	12.46	62	5000	15
**$Cells_L$**	0.14	0.7	5000	15
**Plastic**	2.6 cm^2	1.6	0.6 pmol/cm2	
**WS conc. DMBT**	330 nM (nominal conc. 1.0 µM)			

**B**	**Withouth BSA (DMT)**			
**compartments**	**Volume [µL]**	**VIVD predicted amount [pmol]**	**VIVD predicted molarity [nM]**	**Predicted conc./WS. conc.**
**Medium**	400	3.5	9	0.04
**$Medium_u$**	400	0.4	1	0.004
**$Cells_t$**	14	94	7000	28
**$Cells_M$**	1.4	9.5	7000	28
**$Cells_{IW}$**	12.46	84	7000	28
**$Cells_L$**	0.14	1	7000	28
**Plastic**	2.6 cm^2	2	0.8 pmol/cm2	
**WS conc. DMT**	250 nM (nominal conc. 1.0 µM)			

Supplementary Figure 9: Tabular overview of VIVD-predicted quantities of TEBU.
Tables show the predicted distribution of TEBU across medium, cellular sub-compartments, and plastic under (A) BSA-containing (DMBT) and (B) BSA-free (DMT) conditions. Amounts (pmol), volumes (µL), and corresponding predicted concentrations (nM) are shown. The last columns indicates the ratio of predicted (VIVD) concentrations to working stock (WS) concentrations. $Cells_t$: total cellular amount; $Cells_M$: mitochondrial compartment; $Cells_{IW}$: intracellular water compartment; $Cells_L$: lysosomal compartment; $Medium_u$: unbound compound in medium. Plastic values are expressed as surface-normalized amounts (pmol/cm^2).

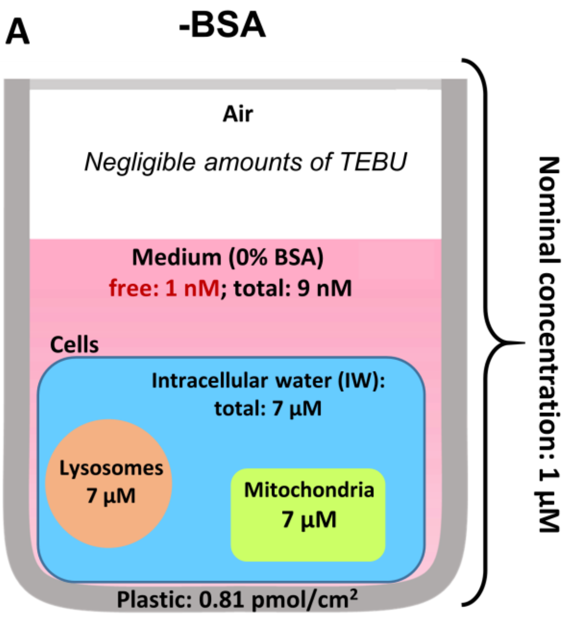

B

	Experiment	d	e	f
	BSA	-	-	+
	nominal concentration [µM]	0.3	1.0	1.0
Recovered quantity in medium	Experimental (E) [pmol]	2	7	128
	Predicted (P) [pmol]	1	3.5	61
	P/E ratio	0.51	0.48	0.47
Recovered quantity in cells	Experimental (E) [pmol]	17	57	44
	Predicted (P) [pmol]	28	95	70
	P/E ratio	1.67	1.66	1.59

Supplementary Figure 10: Predicted distribution of TEBU in cell cultures and comparison of predicted vs. experimental data.

(A) Schematic illustration of the VIVD-predicted distribution of TEBU in a cell culture well under BSA-free condition (DMT). Partitioning between medium, intracellular water (IW), mitochondria, lysosomes, and plastic is shown at a nominal concentration of 1 µM. The air compartment was assumed to contain no TEBU due to its negligible volatility. (B) Comparison of experimentally recovered (E) and VIVD-predicted (P) amounts of TEBU in medium and cells across different conditions (d–f) with or without BSA (see details on conditions d-f in Figure S5). Nominal concentrations were 0.3 µM (d) and 1.0 µM (e, f). This figure supports and provides additional detail for Figure 8 in the main text.

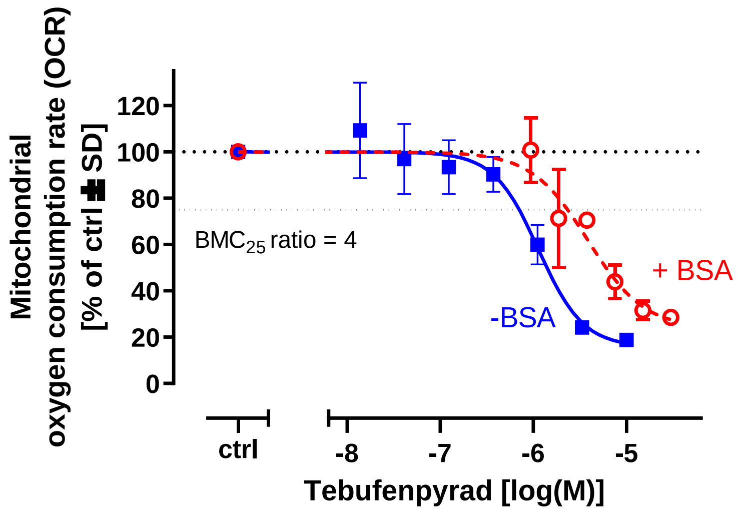

Supplementary Figure 11: Effect of BSA on TEBU-induced inhibition of mitochondrial respiration in LUHMES cells.

The mitochondrial oxygen consumption rate (OCR) was measured in LUHMES cells cultured under NeuriTox-M (galactose) assay condition, following acute exposure to TEBU, in the absence (0% BSA) or presence (1% BSA) of bovine serum albumin (BSA). Data are expressed relative to the DMSO (0.1%) solvent control (ctrl) as means ± SD of two biological replicates. The ratio BMC25 (BSA 1%)/ BMC25 (BSA 0%) is used to quantify the effect of the BSA on toxicity.

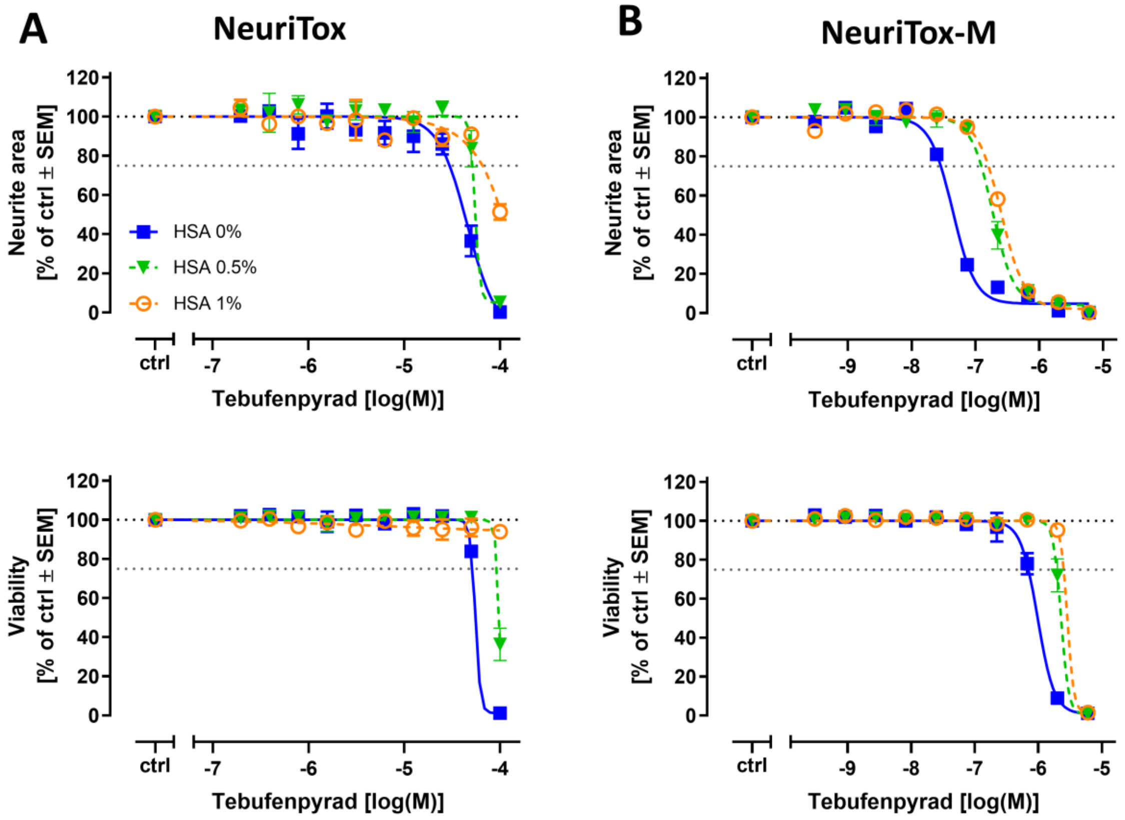

Supplementary Figure 12: Effect of human serum albumin (HSA) on TEBU-induced neurotoxicity.

LUHMES cells were treated from d2 to d3 for 24 h with TEBU under (A) NeuriTox and (B) NeuriTox-M assay conditions, in the presence of increasing concentrations of human serum albumin (HSA: 0%, 0.5%, and 1%). Then cells were stained with calcein-AM and Hoechst H-33342, and imaged using the Cellomics CellInsight microscope. Neurite area (NA) and cell viability (V) were assessed using an automated algorithm and are shown relative to the DMSO (0.1%) solvent control (means ± SEM, *n* = 3). The BMC25 values for the neurite area is the NeuriTox-M assay were 30 nM (no HSA), 120 nM (0.5% HSA), and 120 nM (1% HSA).

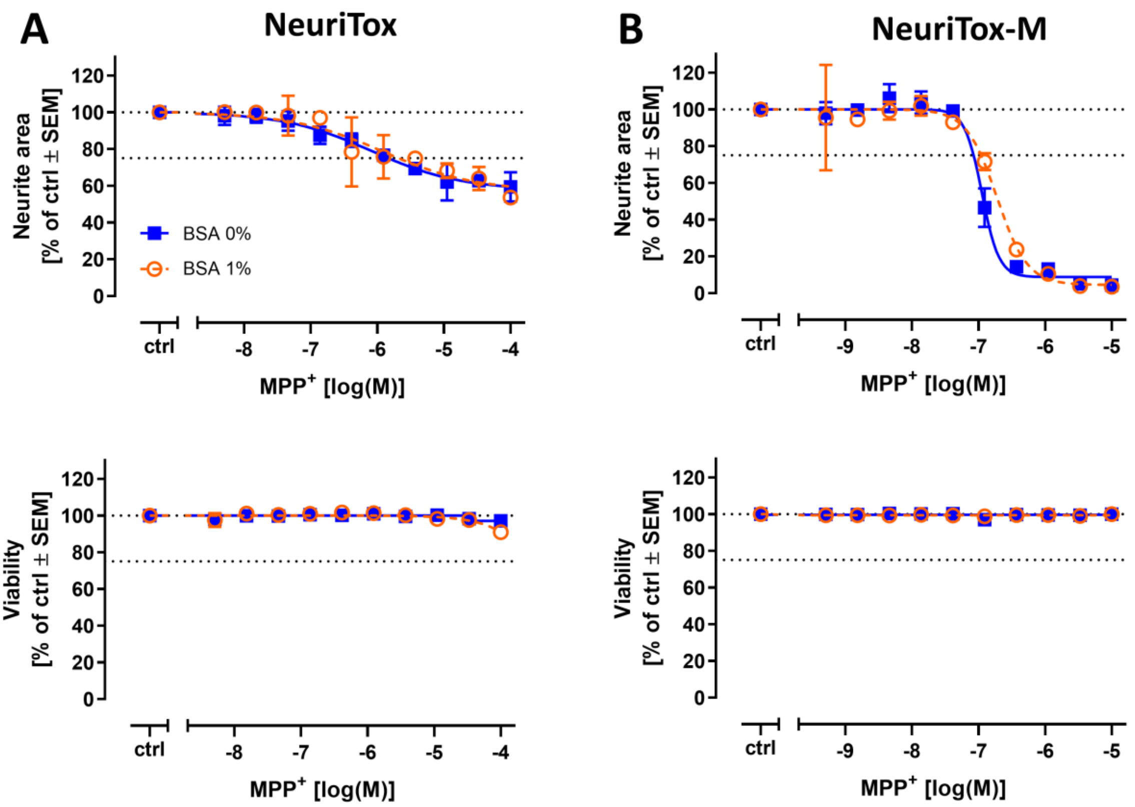

Supplementary Figure 13: Assessment of the potential modulation of MPP^{+} toxicity in LUHMES cells, using the NeuriTox and NeuriTox-M assays.

LUHMES cells were treated from d2 to d3 for 24 h with MPP^{+} under (A) NeuriTox and (B) NeuriTox-M assay conditions, with and without 1% BSA. Then cells were stained with calcein-AM and Hoechst H-33342, and imaged using the Cellomics CellInsight microscope. Neurite area (NA) and cell viability (V) were assessed using an automated algorithm and are shown relative to the DMSO (0.1%) solvent control (data are means ± SEM, *n*=3).

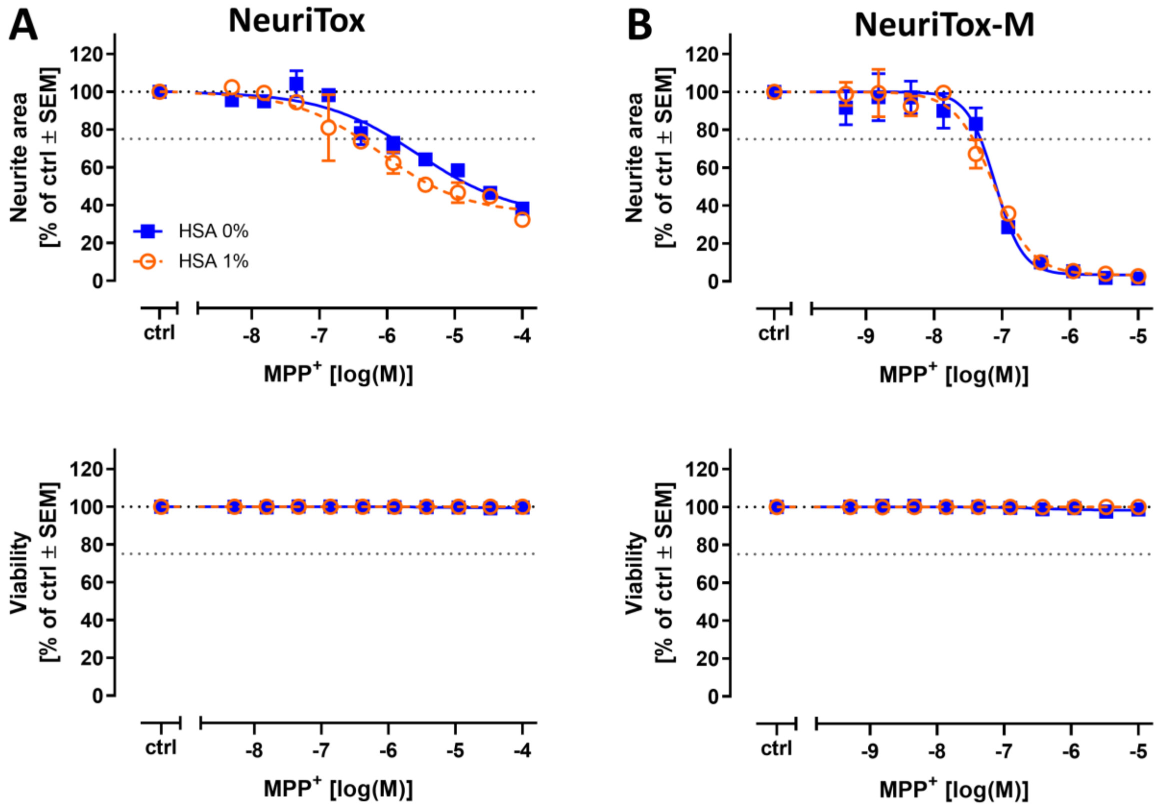

Supplementary Figure 14: Effect of human serum albumin (HSAF) on MPP$^+$ induced neurotoxicity.

LUHMES cells were treated from d2 to d3 for 24 h with MPP$^+$ under (A) NeuriTox and (B) NeuriTox-M assay conditions, with and without 1% HSA. Then cells stained with calcein-AM and Hoechst H-33342, and imaged using the Cellomics CellInsight microscope. Neurite area (NA) and cell viability (V) were assessed using an automated algorithm and are shown relative to the DMSO (0.1%) solvent control (means ± SEM, *n*=3).

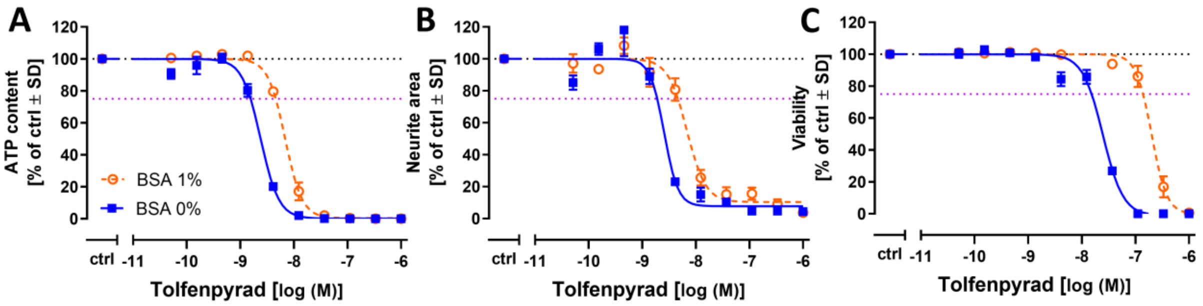

D	1% BSA	ATP BMC$_{25}$ [nM]	NA BMC$_{25}$ [nM]	V BMC$_{25}$ [nM]	Toxicity ratios of plus vs minus BSA data		
					ATP	NA	V
Direct injection (DI)	-	1.5	2	15	3	2.5	9
	+	5	5	140			

Supplementary Figure 15: Tolefenpyrad toxicity under modified handling condition in the absence and presence of 1% BSA.

(A–C) Concentration–response curves for ATP content, neurite area, and cell viability under NeuriTox-M assay. In the standard setup, concentrations were generated by serial dilution in the master block prior to transfer to cells. In the modified approach (direct injection (DI)), each concentration was prepared independently in DMSO, then diluted in medium (1% DMSO), and added directly to cells without serial dilution (0.1% DMSO). Cells were seeded in 60 µL medium per well, and 20 µL of compound solution was added to reach final concentrations. (D) Corresponding BMC25 values and toxicity ratios comparing conditions with and without BSA. Data are expressed relative to the DMSO (0.1%) solvent control (ctrl) and presented as mean ± SD.

Supplementary References

1. Sharma, N.; Mandal, K.; Sharma, S. Validation of LCMS/MS based method for residual estimation of tolfenpyrad and emamectin benzoate in brinjal and cauliflower and their risk assessment. *Journal of Food Composition and Analysis* **2024**, *136*, 106803, doi.org/10.1016/j.jfca.2024.106803.
2. Wang, Z.H.; Wang, X.R.; Wang, M.; Li, Z.Q.; Zhang, X.Z.; Zhou, L.; Sun, H.Z.; Yang, M.; Lou, Z.Y.; Chen, Z.M.; et al. Establishment of a QuEChERS-UPLC-MS/MS Method for Simultaneously Detecting Tolfenpyrad and Its Metabolites in Tea. *Agronomy-Basel* **2022**, *12*, 2324, doi.org/10.3390/agronomy12102324.
